# NAD^+^ reverses Alzheimer’s neurological deficits via regulating differential alternative RNA splicing of *EVA1C*

**DOI:** 10.1126/sciadv.ady9811

**Published:** 2025-11-07

**Authors:** Ruixue Ai, Lipeng Mao, Xurui Jin, Carlos Campos-Marques, Shi-qi Zhang, Junping Pan, Maria Jose Lagartos-Donate, Shu-Qin Cao, Beatriz Barros-Santos, Rita Nóbrega-Martins, Filippos Katsaitis, Guang Yang, Chenglong Xie, Xiongbin Kang, Pingjie Wang, Manuele Novello, Yang Hu, Linda Hildegard Bergersen, Jon Storm-Mathisen, Hidehito Kuroyanagi, Beatriz Escobar-Doncel, Noemí Villaseca González, Farrukh Abbas Chaudhry, Zeyuan Wang, Qiang Zhang, Guang Lu, Ioannis Sotiropoulos, Zhangming Niu, Guobing Chen, Rajeevkumar Raveendran Nair, Joana Margarida Silva, Oscar Junhong Luo, Evandro Fei Fang

**Affiliations:** ^1^ Department of Clinical Molecular Biology, University of Oslo and Akershus University Hospital, 1478 Lørenskog, Norway.; ^2^ Department of Oncology, The First Affiliated Hospital, School of Medicine, Jian University, 510632 Guangzhou, China.; ^3^ Guangdong–Hong Kong–Macau Great Bay Area Geroscience Joint Laboratory, 510632 Guangzhou, China.; ^4^ Department of Systems Biomedical Sciences, School of Medicine, Jinan University, 510632 Guangzhou, China.; ^5^ Mindrank AI Ltd., Hangzhou, China.; ^6^ Life and Health Sciences Research Institute (ICVS), School of Medicine, University of Minho, Campus Gualtar, 4710-057 Braga, Portugal.; ^7^ ICVS/3B’s - PT Government Associate Laboratory, Braga/Guimarães, Portugal.; ^8^ Department of Microbiology and Immunology, Institute of Geriatric Immunology, School of Medicine, Jinan University, 510632 Guangzhou, China.; ^9^ Laboratory of Brain Exosomes and Pathology – ExoBrain, Institute of Biosciences and Applications, National Center for Scientific Research “Demokritos,” T. Patriarchou Grigoriou & Neapoleos, 15310 Athens, Greece.; ^10^ Bioengineering Department and Imperial-X, Imperial College London, London W12 7SL, UK.; ^11^ National Heart and Lung Institute, Imperial College London, London SW7 2AZ, UK.; ^12^ Cardiovascular Research Centre, Royal Brompton Hospital, London SW3 6NP, UK.; ^13^ School of Biomedical Engineering and Imaging Sciences, King’s College London, London WC2R 2LS, UK.; ^14^ Department of Neurology, The First Affiliated Hospital of Wenzhou Medical University, Wenzhou, 325000 Zhejiang, China.; ^15^ Institute Of Aging, Wenzhou Medical University, Wenzhou, 325000 Zhejiang, China.; ^16^ Genome Data Science, Faculty of Technology, Bielefeld University, Bielefeld, Germany.; ^17^ Department of Geriatrics, The First Affiliated Hospital, Zhengzhou University, 450052 Zhengzhou, China.; ^18^ Brain and Muscle Energy Group–Preclinical Laboratories, University of Oslo, 0316 Oslo, Norway.; ^19^ Centre of Excellence KAUST Smart Health, King Abdullah, University of Science and Technology, Thuwai 23955-6900 Kingdom of Saudi Arabia.; ^20^ Department of Molecular Medicine, Institute of Basic Medical Sciences, University of Oslo, NO-0317 Oslo, Norway.; ^21^ Department of Biochemistry, University of the Ryukyus Graduate School of Medicine, Ginowan, Okinawa 901-2720, Japan.; ^22^ Faculty of Pharmacy and Biomedicine Institute, University of Castilla–La Mancha, 02008 Albacete, Spain.; ^23^ Department of Pathology, Oslo University Hospital – Rikshospitalet, Oslo, Norway.; ^24^ College of Computer Science and Technology, Zhejiang University, Hangzhou 310027, China.; ^25^ Hangzhou Global Scientific and Technological Innovation Center, Zhejiang University, Hangzhou 311200, China.; ^26^ Zhongshan School of Medicine, Sun Yat-sen University, Guangzhou, China.; ^27^ Kavli Institute for Systems Neuroscience, NTNU, Fred Kavli-bygget, Olav Kyrres gate 9,7030 Trondheim, Norway.; ^28^ The Norwegian Centre on Healthy Ageing (NO-Age) and The Norwegian National Anti-Alzheimer’s Disease (NO-AD) Networks, 0372 Oslo, Norway.

## Abstract

Dysfunctional alternative splicing events (ASEs) in RNA are markers of aging and Alzheimer’s disease (AD). As a key neuronal resilience metabolite, the oxidized nicotinamide adenine dinucleotide (NAD^+^) slows down AD progression in preclinical studies with several clinical trials ongoing. However, the underlying molecular mechanisms around how NAD^+^ enhances neuronal resilience, especially whether it has any effect on ASEs, have remained elusive. This study shows that NAD^+^ augmentation corrects the ASEs of many genes via a key protein, EVA1C (epithelial V-like antigen 1 homolog C), which is involved in neuronal development and activities. EVA1C is reduced in the hippocampus in patients with AD compared to cognitively normal ones. NAD^+^-induced memory retention is partially dependent on EVA1C, as adeno-associated virus–based *Eva1c* knockdown in the hippocampal CA1 region annuls NAD^+^-induced memory improvement in pathological Tau–bearing mice. We propose that NAD^+^ reduces AD pathologies, at least partially, via amplification of the NAD^+^-*EVA1C* splicing axis, pointing to a potential splice-switching therapy for AD.

## Teaser

A mechanism describing how NAD^+^ regulates alternative RNA splicing to inhibit Alzheimer’s disease is unveiled.

## INTRODUCTION

Dementia is a prevalent and devastating disease that is reported by the World Health Organization to be the seventh leading cause of mortality worldwide (*1*). Alzheimer’s disease (AD) is the most common form of dementia with no cure at the moment (*1*). Intracellular neurofibrillary tangles (NFTs), composed of hyperphosphorylated Tau (pTau) proteins, are a disease-defining neuropathological feature in the AD brains (*2*, *3*). With decades of intensive studies of AD etiologies, it is generally believed that AD is not a homogeneous condition but a group of heterogeneous disease with potential causes and risks ranging from mutations (*APP*, *PS1*, and *PS2*), genetic risks (e.g., *APOE4*), aging, inflammation, senescence, compromised autophagy/mitophagy, damaged mitochondria, and defective alternative splicing events (ASEs) (*4*–*7*).

ASEs represent an important posttranscriptional regulatory mechanism that affects 92 to 94% of human genes (*8*). ASEs are unique to eukaryotic cells—a process of removing introns and assembling exons to construct multiple RNA transcript isoforms from a single pre-mRNA transcript (*9*). The function of one transcript isoform can be related to, distinct from, or even opposite to the function of other transcript isoforms from the same gene (*8*, *10*, *11*). The processes that regulate alternative splicing are abundant and conserved in the vertebrate nervous system (*12*), resulting in phenotypic differences between individual cells or organisms. Aberrant mRNA ASEs are associated with AD (*13*, *14*) and have been documented in candidate AD-associated genes including murine and human microtubule-associated protein Tau (*MAPT*), which encodes the different Tau isoforms (*14*, *15*). The exon 3–containing *MAPT* mRNA transcripts decrease the aggregation of NFTs (*14*, *15*). However, analyses of the AD brain proteomic profiles identified structural changes in insoluble U1 small nuclear ribonucleoprotein (snRNP) particle, a component of the spliceosome (*16*). Furthermore, a global transcriptome association study of 450 subjects in two aging cohorts identified hundreds of ASEs reproducibly associated with AD pathology (*17*). Additional studies support the idea that ASEs are an important feature of AD brains and that ASEs are, in some cases, modulated by genetic and/or protein factors (*17*).

Nicotinamide adenine dinucleotide (oxidized form) (NAD^+^) plays a fundamental role in life and health including in brain protection (*18*, *19*). Emerging evidence suggests that age- and gene-dependent NAD^+^ depletion and/or impaired NAD^+^-dependent pathways play a role in AD pathophysiology (*20*, *21*). In rodent models of early-onset familial AD, NAD^+^ depletion and NAD^+^-associated metabolic dysfunction have been linked to disease pathology in brain tissue (*22*). Meanwhile, strategies in NAD^+^ augmentation, such as via supplementing its precursors nicotinamide riboside (NR) or nicotinamide mononucleotide (NMN), inhibited pTau and improved cognitive function in a cross-species study involving a neuronal Tau (proaggregate F3ΔK280 Tau fragment) transgenic *Caenorhabditis elegans* model (*21*), triple-transgenic AD (3×Tg AD)/DNA polymerase β heterozygous knockout (Polβ^+/−^) mice (*23*), and 3×Tg AD mice (*24*). However, only a small number of studies have addressed the role of ASEs in tauopathies or other AD-related pathologies (*25*), and a comprehensive mechanistic study of the relationships between NAD^+^ metabolism and ASEs in tauopathies is lacking.

In 2016, the US Food and Drug Administration approved therapeutic use of small-molecule compounds and genomics-based technologies including oligonucleotides via direct delivery into the brains of patients afflicted by central nervous system (CNS) diseases (*26*). This type of therapy has been tested preclinically for refractory neurological disorders (*26*) including erythropoietic protoporphyria (*27*), cystic fibrosis (*28*), congenital deafness (*29*), and cancers such as glioblastoma (*30*), prostate (*31*), and breast cancer (*32*). However, the development and application of therapeutics that target mRNA splicing have been challenging, at least in part because the biological effects of ASEs are complex and difficult to predict (*27*). Therefore, there is an urgent need for a better understanding of the regulation and downstream effects of ASEs, especially in the context of AD, tauopathies, and related neurological diseases, with a final goal of developing effective therapies.

Epithelial V-like antigen 1 homolog C (EVA1C) is an immunoglobulin superfamily protein that plays an important role in neuronal development and function, while its role in AD is obscure (*33*, *34*). To understand the biological role of *Eva1c* and its isoforms in mice with or without a tauopathy phenotype, it is important to identify and understand EVA1C protein-protein interactions (PPIs). Several high-throughput methods for analyzing PPIs are available including yeast two-hybrid screens (*35*), tandem affinity purification (*36*), and mass spectrometric protein complex identification (*37*); however, these methods are time consuming and prone to high false-positive and false-negative rates (*37*, *38*). Recent analyses of PPIs use advanced computer-driven artificial intelligence (AI)–based deep learning algorithms to predict PPIs. One such approach is DeepPPI, which deduces high-level protein features from protein sequence data and has outperformed traditional machine learning algorithms (*39*). However, currently available methods use protein amino acid sequences (primary structure) as input and therefore may neglect important features of protein secondary and tertiary structures and three-dimensional (3D) conformations (*40*–*43*). The present study uses a integrative approach, whereby genetic variation, predicted 3D protein structures, and predicted PPIs are modeled using large-scale bioinformatic analyses and deep learning algorithms. The output data are then verified in a “wet” lab–based cross-species experimental system. Using this approach, we performed global transcriptomic analyses and identified ASEs in RNA sequencing (RNA-seq) data from the hippocampi of 14-month-old wild-type (WT) and hTau.P301S mice with or without exposure to NR. Focusing on *EVA1C*, a deep learning framework was used to predict EVA1C protein structures and PPIs, its mechanism(s) of action, and EVA1C isoform–specific binding affinities to BAG1 (BCL2-associated athanogene 1) and HSP70 (heat shock protein 70) in the presence or absence of NAD^+^ precursors. Our findings were experimentally verified in mammalian cells, nematodes, mice, and postmortem brain tissues. This comprehensive approach yielded insight into the effects of NAD^+^ on ASEs in tauopathies and the potential mechanism(s) of action of EVA1C. The results will inform and improve the future design of mRNA splicing–targeted therapeutics and may facilitate successful future clinical use of these therapeutics.

## RESULTS

### Dysregulation of mRNA splicing in transgenic hTau.P301S mice and aged worms

**Fig. 1.**
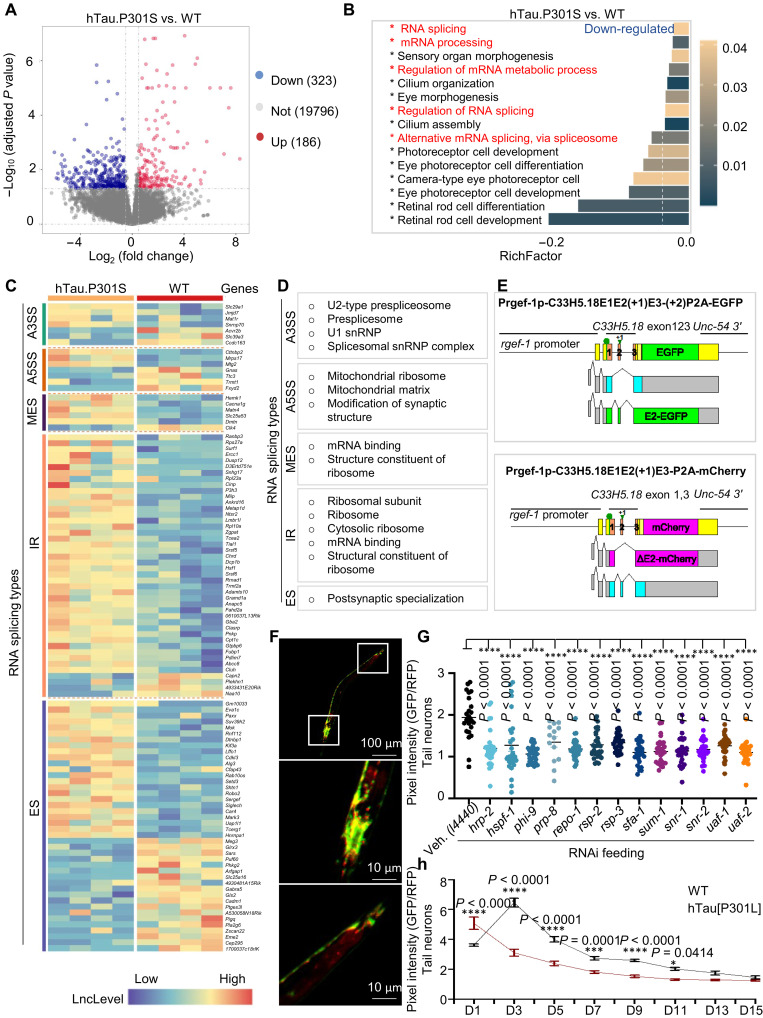
Compromised mRNA splicing in Tau pathology and aging. (**A**) Volcano plot comparing hippocampal RNA-seq–derived gene expression in hTau.P301S and WT mice. Gray dots represent 19,796 genes expressed at similar levels in both strains, red dots represent differentially up-regulated genes, and blue dots represent differentially down-regulated genes in mutant relative to WT mice. (**B**) Top 15 GO pathways enriched (adjusted *P* < 0.05) among genes down-regulated in hTau.P301S versus WT mice; GO terms shown in red relate to mRNA splicing or processing. The criterion for differentially expressed genes (DEGs) is [log_2_(fold change) (log_2_FC) > 0.5 and FDR < 0.05]. (**C**) Heatmaps showing distribution and relative frequency of ASEs stratified by ASE subtype in hTau.P301S versus WT mice; ASE subtypes are alternative 3′ splice site (A3SS), alternative 5′ splice site (A5SS), multiple exon skip (MES), intron retention (IR), and exon skip (ES). (**D**) GO terms enriched in DEGs stratified by ASE subtype. (**E**) Schematic diagram of the *rgef-1* splicing reporter gene cassette. (**F**) Pan-neuronal *rgef-1* splicing in day 1 (D1) *C. elegans*. Inset: Positive control reporter cassette (without frameshifts). Images were captured at ×10 magnification. (**G**) Quantification of heterogeneous pan-neuronal splicing in adult day 3 RNAi-treated versus control worms. Veh., vehicle control. (**H**) Pan-neuronal splicing index in hTau[P301L] versus WT control worms. Data are means ± SEM of pooled total animals from three biological replicates (*n* = 20 to 35 nematodes per group). Differences between conditions were assessed by one-way analysis of variance (ANOVA), while differences between genes or proteins were assessed by two-tailed *t* tests (95% confidence interval). *P < 0.05, ***P < 0.001, and ****P < 0.0001. The top 15 statistically significant up-regulated GO terms [related to (B)] are shown in fig. S1A. A set of representative images of pan-neuronal splicing patterns [related to (G)] is included in fig. S1 (B to R).

The effects of NR on ASEs and the RNA transcriptome were investigated in hippocampal tissue from 14-month-old hTau.P301S and WT mice (control). Eleven-month-old mutant and WT mice were maintained with or without NR, a precursor of NAD^+^, for 3 months, a period that overlaps the age of expected onset of cognitive impairment and neuronal degeneration in hTau.P301S mice. RNA-seq analyses revealed differential up- or down-regulation of 509 genes [false discovery rate (FDR) < 0.05] in hTau.P301S mice relative to WT mice (Fig. 1A and table S1). Gene Ontology (GO) analyses showed that down-regulated genes were enriched especially for RNA processing–related GO biological process and cellular component terms including RNA splicing (richFactor = −0.028), mRNA processing (richFactor = −0.030), regulation of mRNA metabolic process (richFactor = −0.038), regulation of RNA splicing (richFactor = −0.044), and alternative mRNA splicing via spliceosome (richFactor = −0.071) (marked in Fig. 1B and table S2). In contrast, up-regulated genes were not enriched at a statistically significant level for GO terms related to RNA splicing (fig. S1A and table S2). This result suggests that dysregulation of mRNA processing, especially RNA splicing, is a hallmark of tauopathy in the humanized hTau.P301S mouse.

ASEs allow a single eukaryotic gene to express functionally distinct proteins (*44*, *45*) and belong to five distinct types: alternative 3′ splice site (A3SS), alternative 5′ splice site (A5SS), multiple exon skip (MES), intron retention (IR), and exon skip (ES). RNA-seq analysis of ASEs in the hTau.P301S transcriptome revealed that IR events were the most frequent ASE type (44 IR events in 106 total ASEs) and ES events were slightly less frequent (*42*), while the number of the remaining three ASE types was much lower (A3SS, 7; A5SS, 7; MES, 6) (Fig. 1C and table S3). Furthermore, GO analysis revealed distinct functional enrichments, such that A3SS, MES, and IR events were enriched in genes involved in RNA metabolic processing (Fig. 1D), while A5SS events were enriched in genes that regulate mitochondrial ribosomes or relate to mitochondrial translation and respiratory system process (Fig. 1D). Some genes carrying A5SS and ES events colocated and regulated synaptic function (Fig. 1D). These results suggest that ASEs, especially A5SS and ES events, in genes related to mitochondrial and synaptic function were dysregulated in hTau.P301S mice.

As ES was a top event of ASE in hTau.P301S mice and its important role in neuronal function, we generated a roundworm *C. elegans* model to monitor neuronal ES event during aging and in Tau pathology. While there are differences of ASE among worms, mice, and humans, at least some of the major ASE pathways are evolutionally conservative. The roundworm *C. elegans* strain KH2566, which carries a pair of pan-neuronally expressed reporter minigenes, was used to examine whether and how aging affects mRNA splicing and ASEs. In KH2566 worms, expression of the *C33H5.18* exon 2 fluorescent reporter minigenes is driven by the neuronal *rgef-1* promoter, and each of the minigenes carries a distinct frameshift mutation. ASEs in the reporter genes are monitored by fluorescence imaging to detect the intensity and ratio of green fluorescent protein (GFP):mCherry fluorescence. Expression of GFP depends on the presence of exon 2, while expression of mCherry occurs when exon 2 is skipped (Fig. 1E). Therefore, the GFP:mCherry ratio is a quantitative measure of ASE events in neurons of KH2566 worms (Fig. 1F). The RNA splicing index (the ratio of green to red fluorescence in KH2566 worms) correlates inversely with the frequency of exon 2 skipping in worms carrying reporter minigenes. To test whether some of the major ASE regulatory pathways are conserved between worms and mammals, we neuron-specifically RNA interference (RNAi)-knocked down worms to a comprehensive set of spliceosome components (Fig. 1G; fig. S1, B to R; and table S4) (*46*). Reduction of these 13 spliceosome components greatly increased exon 2 skipping, as evidenced by reduced GFP/red fluorescent protein (RFP) ratios (Fig. 1G). These data indicate a likelihood of conservation (at least partially) of the ASE machinery between worms and mammals, and our KH2566 reporter gene system is an effective tool for monitoring the fidelity of endogenous mRNA splicing in the neurons of living *C. elegans*. Thus, we used this reporter gene system to study the linkages between aging (and AD) in the fidelity of mRNA splicing and with any findings to be validated in human cellular systems. To use this reporter gene system in worms with tauopathy-like disease, we crossed KH2566 worms with CK12, hTau[P301L] worms (*47*, *48*), a well-characterized strain expressing pan-neuronal human Tau (hTau) 4R1N P301L that mimics tauopathy-like disease including impaired memory functions (*21*, *49*).

Pre-mRNA splicing is tightly regulated during *C. elegans* development (*50*, *51*), and studies using an mRNA splicing reporter gene suggest that some developmentally regulated ASEs are expressed in a tissue-specific manner (*52*). In hTau[P301L] worms carrying the reporter minigenes described above, exon 2 skipping (ES) events increase consistently from adult day 1 to day 3 (will be simplified to “day x” onward) with age, while in WT worm, exon 2 skipping decreases from day 1 to day 3 before beginning to increase consistently with age (Fig. 1H). Notably, fewer exon 2 skipping events were observed in adult day 1 hTau[P301L] worms than in WT worms, whereas this relationship reversed, and more exon 2 skipping events were observed in hTau[P301L] worms than in WT worms after day 3 (Fig. 1H). We observed that the splicing index in WT is nearly twofold higher than in hTau[P301L] worms on day 3, but the difference decreased gradually from day 3 to day 13, when the differences were smaller (Fig. 1H). These results suggest that neuronal RNA splicing homeostasis is maintained in young hTau[P301L] worms by a compensatory mechanism. The splicing index showed different trends in the gut than in the CNS of WT and hTau[P301L] worms (fig. S1, S to U), indicating that the compensatory mechanism in neurons is a tissue-specific phenomenon.

### NR up-regulates genes involved in RNA splicing and components of the spliceosome in tauopathy

Previous studies show that mRNA processing is modulated by NAD^+^ in viruses, yeast, bacteria, and humans. Notably, mRNA splicing is perturbed in patients with AD resulting in alterations to splicing signals, trans-acting regulatory factors, and components of the splicing machinery (*53*–*59*). Furthermore, a recent study suggests that aberrant RNA splicing processes contribute substantially to pathophysiology in the PS19 transgenic mouse model of tauopathy (*25*). Here, the effects of NR on RNA splicing were investigated in worm and mammalian cell–based tauopathy models.

**Fig. 2.**
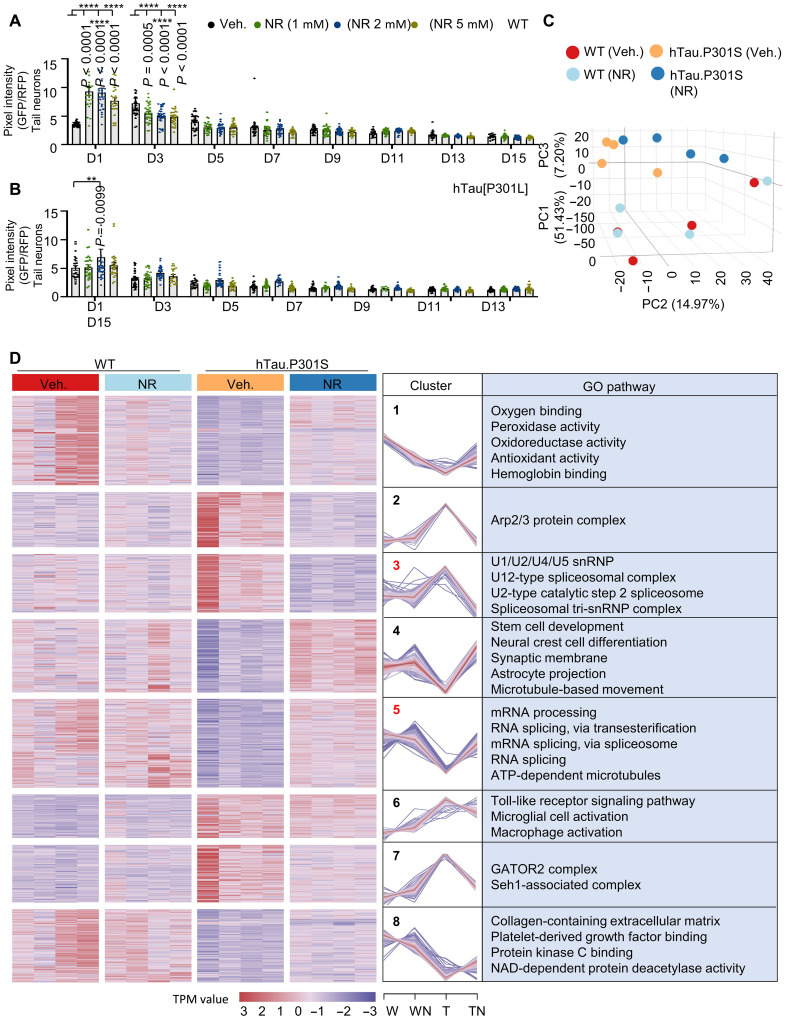
NR normalizes gene expression in tauopathy worms. (**A** and **B**) Pan-neuronal RNA splicing index in day 1 to day 13 worms treated with 0, 1, 2, or 5 mM NR: WT (A) and hTau[P301L] (B). Vehicle control data are for one set of experiments in Fig. 1H. (**C**) 3D graph of principal components analysis (PCA) analysis of mouse hippocampal gene expression in WT (Veh.), WT (NR), hTau.P301S (Veh.), and hTau.P301S (NR) mice. (**D**) Heatmaps show up- and down-DEGs in four experimental groups of mice as indicated (left). Eight clusters of DEGs (left) are represented graphically (middle). GO analysis of DEGs stratified by gene cluster (right). The criterion for statistical significance was adjusted *P* < 0.05. GO terms shown in red are related to mRNA splicing or the spliceosome. W, WT; WN, WT with NR; T, hTau.P301S; TN, hTau.P301S with NR; Arp2/3, actin-related protein 2/3 complex; GATOR2, GAP activity toward Rags complex 2; Seh1, SEH1-like nucleoporin. Data are means ± SEM of pooled total animals from three biological replicates (*n* = 20 to 35 nematodes per group). One-way ANOVA was used to assess statistical significance. **P < 0.01 and ****P < 0.0001.

WT and hTau[P301L] worms were treated with NR (1, 2, and 5 mM) or vehicle (control) from egg hatching on day 1 to day 15 and were monitored for changes in fluorescence, and the splicing index was calculated for each experimental group. The splicing index increased more than twofold in NR-treated 1-day-old WT worms relative to vehicle-treated worms, but by day 3, this increase changed to ~25% reduction compared to vehicle-treated controls (Fig. 2A). In contrast, 2 mM NR only increased the RNA splicing index by 20% in 1-day-old hTau[P301L] worms, and the splicing index was not altered by NR in any other tested group of hTau[P301L] worms (Fig. 2B). These results suggest that NAD^+^ may only regulate RNA splicing during the earliest stage of worm development.

RNA-seq data from the hippocampal region of mouse brains were used to determine whether and how NR influences mRNA transcription and splicing in hTau.P301S and WT mice. We treated both WT and hTau.P301S mice with NR (6 mM in drinking water), as described (*60*) for 2 months, followed by hippocampal tissue collection for RNA-seq. To identify general similarities in the RNA-seq datasets, we conducted principal components analysis (PCA) comparing hTau.P301S with or without NR to WT mice with or without NR. Results of PCA showed a clear separation between transcriptional patterns in WT and hTau.P301S transgenic mice (Fig. 2C and table S5). PCA also detected two subgroups of NR-treated hTau.P301S mice (Fig. 2C) but only partial separation between NR-treated and vehicle-treated WT mice (Fig. 2C). These data suggest that mRNA transcription and splicing in the hippocampal brain region may be different markedly between hTau.P301S and WT mice, possibly with hTau.P301S mice being more sensitive than WT mice to the effects of NR.

RNA-seq data were then used to identify genes and signaling pathways that were differentially expressed in hTau.P301S or WT mice with or without NR. This analysis identified 730 up- or down-regulated differentially expressed genes (DEGs) (adjusted *P* < 0.05), shown graphically with a heatmap in Fig. 2D (left) (table S6). These DEGs align into eight clusters that show different temporal expression patterns (Fig. 2D, middle, and table S6). Functional enrichment analysis revealed functions related to RNA splicing in cluster 3 including four categories strongly related to the spliceosome, a large RNA-protein complex critical for pre-mRNA splicing. These categories were U1/U2/U4/U5 snRNP, U12-type spliceosome complex, U2-type catalytic step 2 spliceosome, and spliceosomal tri-snRNP complex (*61*). Cluster 5 includes three functions directly related to pre-mRNA splicing and one related to adenosine 5′-triphosphate (ATP)–dependent microtubule motor activity. The expression patterns were oppositely regulated in clusters 3 and 5, with 79 genes in cluster 3 up-regulated in hTau.P301S mice relative to WT and then rapidly down-regulated with NR treatment, while 120 genes in cluster 5 were down-regulated in hTau.P301S mice relative to WT but strongly up-regulated with NR. Notably, in WT mice, genes in clusters 3 and 5 were neither up- nor down-regulated by NR (Fig. 2D, right, and table S6). These results suggest that genes directly involved in RNA splicing were down-regulated in tauopathies. Furthermore, we propose that a compensatory mechanism induces the spliceosome in the absence of NR, but this is reversed in the presence of NR. As a result, compensatory up-regulation of spliceosome-related genes is absent in the hippocampal brain region of NR-treated hTau.P301S mice.

### Effect of NR on global transcriptome structure in tauopathy and WT mice

**Fig. 3.**
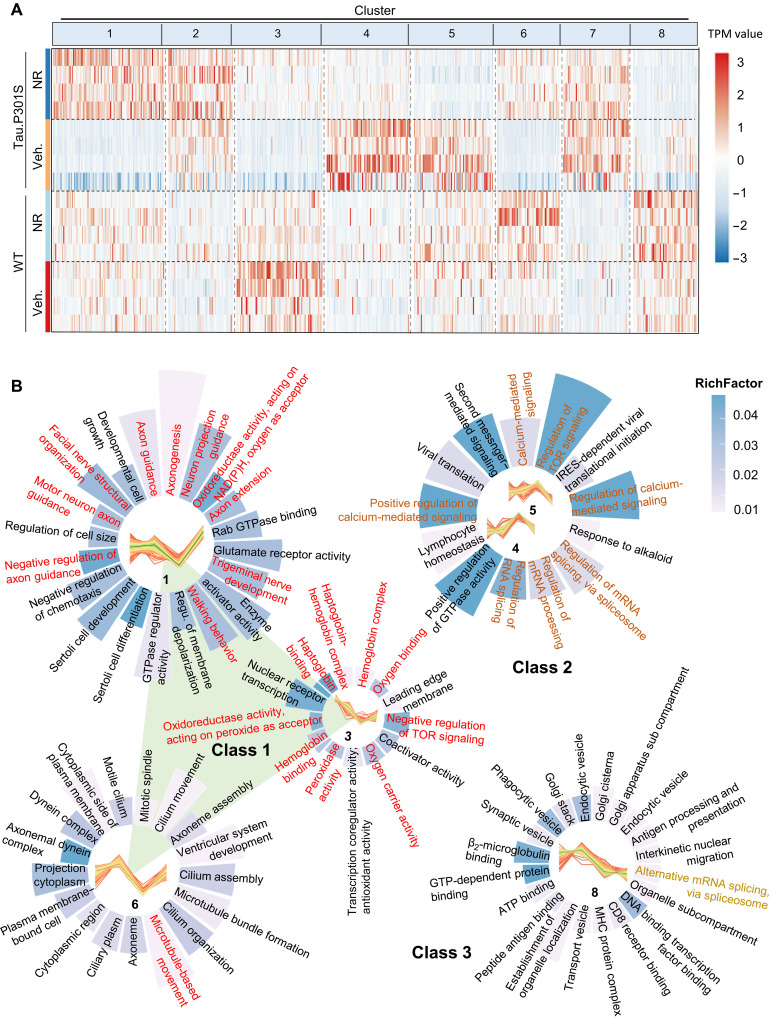
NR induces transcription of genes involved in axon development, oxygen metabolism, mitochondrion localization, and autophagy in tauopathy mice. (**A**) Heatmaps showing DEGs and eight classes of DEG derived by c-means cluster analysis for the four experimental groups of mice as indicated. The scale of relative differential expression is shown to the right, where red corresponds to maximum up-regulation and blue corresponds to maximum down-regulation of gene expression. (**B**) GO terms enriched in each of the eight classes of clusters in (A); each circular bar graph represents one cluster or class of clusters, and bar length correlates with the number of DEGs represented. GO terms shown in red font relate to mRNA. Clusters 4 and 5 are similar and are grouped together. Class 1 includes clusters 1, 2, and 6 marked with red. Class 2 includes clusters 4 and 5 marked with orange. Class 3 includes cluster 8 marked with yellow. GTPase, guanosine triphosphatase; NAD(P)H, reduced form of NAD phosphate; IRES, internal ribosomal entry site; GTP, guanosine 5′-triphosphate; MHC, major histocompatibility complex.

Figure 2 (C and D) shows that Tau pathology polarizes the global transcriptome such that hTau.P301S and WT transcriptomic data map to opposite ends of the principal component 1 (PC1) and PC2 axes (Fig. 2C). NR remodels these relationships, which is consistent with the idea that NR modulates mRNA metabolism in tauopathy mice (Fig. 2D). This observation was explored further in the following experiments, which focus on quantitative expression of genes with open reading frames (see Materials and Methods for details) and the mechanisms underlying NR-dependent changes in patterns of gene expression. This analysis identified eight clusters (Fig. 3A and table S7) with the most significant changes in expression profiles in transgenic hTau.P301S mice. These eight clusters were further grouped into three classes as follows: class 1 clusters 1, 3, and 6 are characterized by up-regulation of transcripts in NR-treated hTau.P301S mice relative to vehicle-treated hTau.P301S mice; class 2 clusters 4 and 5 are characterized by down-regulation of transcripts in NR-treated hTau.P301S mice relative to vehicle-treated hTau.P301S mice; class 3 included only cluster 8 and showed no transcriptomic changes between NR-treated and vehicle-treated hTau.P301S mice (Fig. 3B and table S8).

Class 1 clusters, where NR stimulated expression of genes that are down-regulated in hTau.P301S mice, were enriched in transcripts from 50 signaling pathways including eight pathways related to nerve development and function. At the gene level, 75% of the genes played roles in axon genesis, extension, and guidance, suggesting possible involvement in the formation of neural circuits and synapse cones. The latter processes could promote the synthesis of amyloid-β (Aβ), pTau, and AD pathogenesis (*62*). A pathway involved in trigeminal nerve development, a putative risk factor for dementia (*63*), is also represented in class 1 clusters. Eight pathways involved in oxygen metabolism were enriched in class 1 clusters. Because functional oxygen metabolism is critical for cognitive function, the affected pathways could play roles in AD-associated synaptic dysfunction, neuronal death, and tissue thinning in critical areas of the brain. Gene expression changes also affected microtubule-facilitated movement, as well as mitochondrion localization and transportation. Proper mitochondrial localization is required for formation of functional neural connections (*64*). In addition, the mammalian target of rapamycin (mTOR) pathway was down-regulated by NR in class 1 clusters, correlating with NR’s role in autophagy/mitophagy induction (*21*, *65*); this could ameliorate AD pathology and symptoms (*66*). Expression of transcripts involved in facial nerve structure and organization was also altered by NR, which could potentially normalize facial and eye movement patterns in patients with AD. Last, NR normalized expression of transcripts related to walking, which can be affected in the early stages of dementia (*67*); this is in line perfectly with our recent findings showing the robust antiataxia capacity of NAD^+^ (*60*), a finding further validated in a clinical trial (*68*). These results suggest that multiple mechanisms and pathways mediate the effects of NR on tauopathic mice, including nerve development, oxygen metabolism, microtubule-facilitated movement, and autophagy (Fig. 3B and table S9).

Class 2 clusters, where NR suppressed expression of genes that are up-regulated in hTau.P301S mice, were enriched in 13 signaling pathways including three pathways related to calcium signaling. Excessive Ca^2+^ in mitochondria can lead to mitochondrial dysfunction, high levels of reactive oxygen species, increased apoptosis, and accelerated progression of AD pathology (*69*). Last, three pathways related to RNA splicing were enriched in class 2 clusters (Fig. 3B and table S10).

Class 3 clusters demonstrated no significant transcriptomic changes in NR-treated versus vehicle-treated mice. On the whole, these data suggest that NR normalizes deficiencies affecting the nervous system, oxygen metabolism, mitochondrial localization, autophagy, behavior, Ca^2+^ abundance, and mTOR signaling in hTau.P301S mice. Thus, mRNA splicing is normalized in NR-treated hTau.P301S mice, and the previously active compensatory mechanism becomes superfluous (Fig. 3B and table S11).

### Overview of genes affected by ASEs and the effect of NR on ASE subtypes and distribution in tauopathy mice

**Fig. 4.**
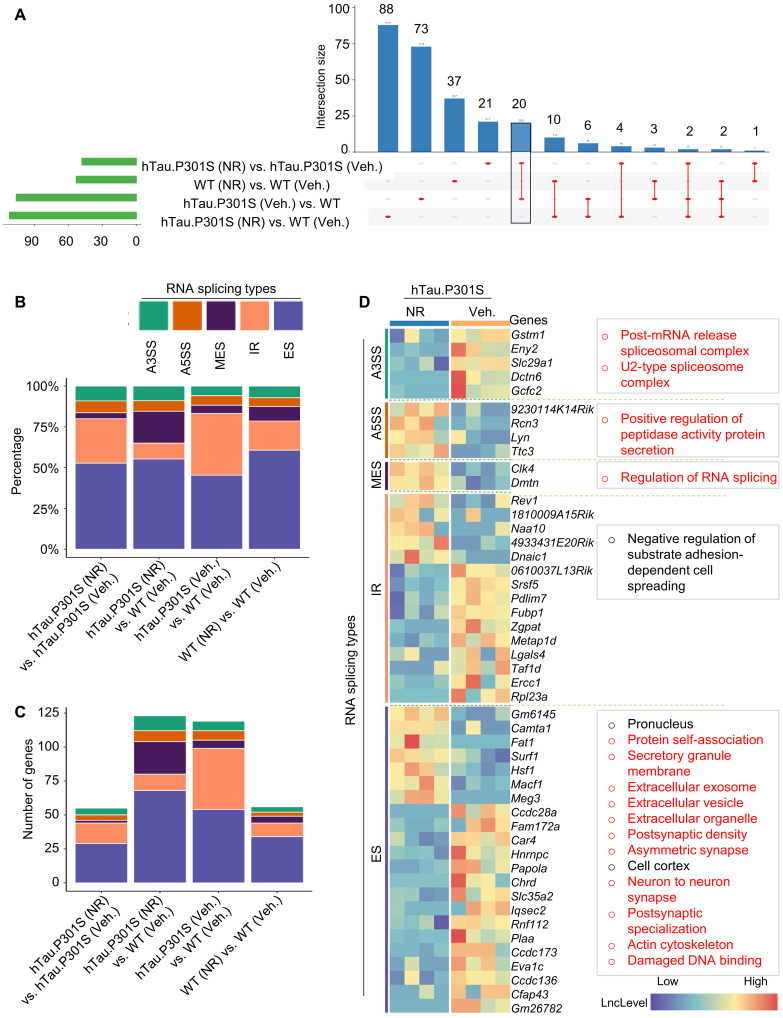
NR ameliorates multidimensional RNA splicing in tauopathy. (**A**) The UpSet plot summarizes the results of six pairwise and two three-way comparisons of ASEs affecting DEGs between hTau.P301S and WT mice treated with or without NR. The intersection size is the number of shared events between the experimental groups. (**B**) For each comparison between experimental groups, the percent representation of each ASE subtype is represented by the height of the corresponding color-coded stacked bar. (**C**) Same as (B), except that bar height represents gene number instead of percentage of total events for each ASE subtype. (**D**) Heatmap comparing DEGs in hTau.P301S transgenic mice in the presence versus the absence of NR, where DEGs are further stratified by ASE subtype. Statistically significant enrichment of GO terms is shown for each DEG subtype. The criterion for statistical significance was adjusted *P* < 0.05. Corresponding data for WT mice are shown in fig. S2.

Alternative mRNA splicing is a critical source of splice-switching targets (*70*, *71*) and is thought to play an important role in AD- and tauopathy-related disease progression. To understand this in greater detail, we determined the number and subtype of ASEs in hTau.P301S and WT mice with or without NR. A total of 267 ASEs were identified as primarily belonging to MES, IR, or ES subtypes (Fig. 4, A to C). Of these, 48 were identified by comparing NR-treated and vehicle-treated hTau.P301S mice, and 21 were specific to NR-treated mice (Fig. 4A and table S12). Fifty-three ASEs were identified by comparing NR-treated and vehicle-treated WT mice, and 37 were specific to NR-treated WT mice (Fig. 4A and table S12). One hundred and six ASEs were identified by comparing vehicle-treated hTau.P301S and vehicle-treated WT mice, and 73 of these were specific to hTau.P301S mice (Fig. 4A and table S12). Twenty ASEs were differentially expressed in vehicle-treated hTau.P301S versus vehicle-treated WT mice and in NR-treated hTau.P301S versus vehicle-treated hTau.P301S mice but not in NR-treated WT versus vehicle-treated WT mice or NR-treated hTau.P301S versus vehicle-treated WT mice. This indicates that treatment with NR selectively suppresses 20 ASEs that are specific to hTau.P301S mice (Fig. 4A and table S12). One of these ASEs occurs in *Eva1c*, whose pattern of expression suggests a role in axon guidance (*34*). This encouraged us to further explore the possible role of *Eva1c* in tauopathy disease progression in the mouse.

We observed that A3SS and A5SS events represented a similar proportion of all ASEs in all experimental groups (Fig. 4B and table S13). In addition, most of ASEs were detected in NR-treated hTau.P301S transgenic mice, indicating that targeting ASEs may be a beneficial effect of NR (Fig. 4C and table S13). Thus, RNA-seq data were mined for ASEs specific to NR-treated hTau.P301S mice (Fig. 4D and table S14). This analysis revealed five gene isoforms affected by A3SS events in NR-treated hTau.P301S mice, including the post-mRNA release spliceosomal complex and the U2-type spliceosome complex. The post-mRNA release spliceosomal complex catalyzes disassembly of the spliceosome separating U2, U5, U6, NineTeen Complex (CPX-1885), and the lariat intron. The U2-type spliceosome complex removes introns from pre-mRNA (*72*). Furthermore, A5SS events were detected in *Riken*, *Rcn3*, *Lyn*, and *Ttc3*, which regulate peptidase activity. This is consistent with earlier reports that peptidase activity is altered in AD (*73*, *74*) and could implicate MES events and altered peptidase activity in AD progression*.* Figure 4B shows that the largest proportion of ASEs and the largest number of genes represented are ES events.

These results also show that ASEs in 22 genes affecting 54 isoforms were disproportionately represented in NR-treated versus vehicle-treated hTau.P301S mice and one of these genes is *Eva1c*. To understand their significance, we performed GO analysis on this subset of genes. This analysis identified 13 core processes and pathways, 11 of which have potential involvement in AD pathophysiology. For example, our data show that NR modulates protein self-association. Protein self-association leads to proteinaceous deposits, Aβ peptide–containing plaques, and NFTs by overcoming unfavorable electrostatic repulsions (*75*, *76*). Furthermore, NR also modulates the expression of genes related to extracellular organelles (i.e., exosomes and vesicles); extracellular organelles carry disease-associated protein aggregates and are linked to AD pathology (*77*). The intracellular secretory granule, which has been reported to release cationic antimicrobial protein of 37 kDa (CAP37), is expressed at a higher level in the brains of patients with AD (*78*, *79*); *CAP37* is a member of this subset of genes. Notably, GO terms related to “synapses” and “actin cytoskeleton” were also enriched in this subset of genes, which may relate to the fact that the actin cytoskeleton regulates the structure and function of dendritic spines (*80*). Genes that promote the response to DNA damage are linked to AD progression (*81*–*83*); NR is also implicated in regulating ES events in these genes (Fig. 4D and fig. S2). These data suggest that NR facilitates multilayered control of ASE events in hTau.P301S mice and that additional research on the underlying mechanism is warranted.

### NR regulates EVA1C expression across species and enhances cognitive function and health span in tauopathy worm via *eva-1*

**Fig. 5.**
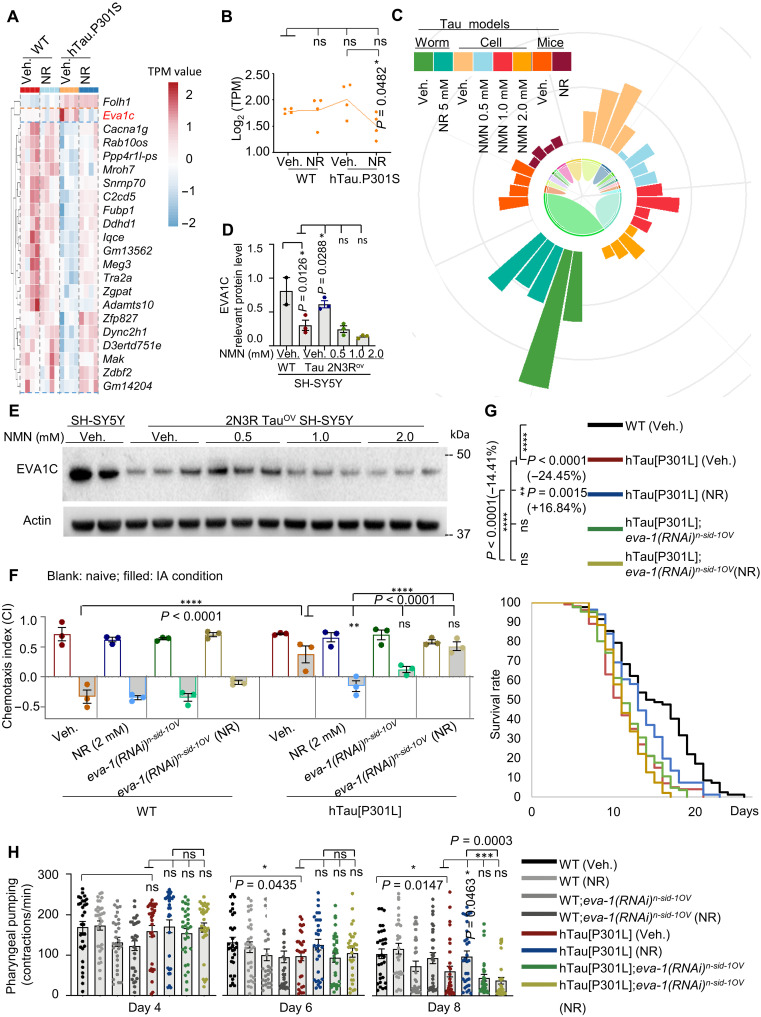
NR-dependent expression of *eva1c* normalizes cognition and extends health span in tauopathy worms. (**A**) Heatmap of open reading frame–containing DEGs affected by ASEs in hTau.P301S and WT mice treated with or without NR. (**B**) Graph shows relative differential expression of *Eva1c* in the four indicated experimental groups. (**C**) Circular bar graph representing expression of *EVA1C* in worms (±NR), human cells (±NMN), and mice (±NR). (**D** and **E**) Immunoblot analysis of EVA1C protein in SH-SY5Y and 2N3R Tau–overexpressing SH-SY5Y cells treated with 0.5, 1.0, or 2.0 mM NMN or vehicle control. Quantification of (E) is shown in (D). (**F**) Chemotaxis index was measured in WT, hTau[P301L], hTau[P301L] worms treated with NR, hTau[P301L] fed with *eva1c* RNAi, and hTau[P301L] fed with *eva1c* RNAi and NR. (**G**) Lifespan was estimated from one set of experiments with three technical replicates (see table S17 for details) and 90 to 120 worms for each biological repeat. Data are shown as means ± SEM. Log-rank test was used to evaluate statistical significance. (**H**) Pharyngeal pumping rate was measured in adult worms (*n* = 30 of pooled total animals from three independent biological repeats) on days 4, 6, and 8. Data of pooled total animals from three biological replicates in (E) (*n* = 20 to 35 nematodes per group) are shown as means ± SEM. One-way ANOVA was used to assess statistical significance. ns, no significance. *P < 0.05, **P < 0.01, ***P < 0.001, and ****P < 0.0001.

To better understand the mechanism(s) by which NAD^+^ regulates RNA splicing, we focused on 22 transcripts whose expression and splicing are modulated by NR in hTau.P301S mice (Fig. 5A and table S15). Because 15 of these transcripts encode proteins, the remaining seven noncoding transcripts were excluded from further study. A comprehensive literature review identified five NR-responsive genes, *Fubp1*, *Zgpat*, *Zfp827*, *Tra2a*, and *C2cd5*, whose primary function appeared to involve binding to DNA, RNA, or other regulatory ions or biomolecules (*84*). Other genes in this group include *Adamts10*, *Zdbf2*, *Cacna1g*, and *Snrnp70*, whose expression patterns are altered in AD (*47*, *85*–*88*). Among the many potential target genes, we picked *Eva1c* for the following mechanistic studies because (i) the expression and splicing of *Eva1c* transcripts were specifically regulated by NR in tauopathy mice (Fig. 4D) and (ii) EVA1C plays a critical role in neuronal function, while the underlying molecular mechanisms remain largely elusive (*34*).

To clarify the roles and contribution of EVA1C to AD pathology and progression, we used several tauopathy models including human SH-SY5Y cells overexpressing 2N3R Tau, hTau[P301L] *C. elegans*, hTau.P301S mice, and human brain tissue. The 2N3R-overexpressing SH-SY5Y cells were chosen because in vitro data indicate that phosphorylated recombinant 2N3R Tau is strongly associated with AD (*89*). We also used two in vivo AD models, hTau[P301L] *C. elegans* and hTau.P301S mice, as well as human brain samples in different Braak stages. The results show that *Eva1c* mRNA is more abundant in vehicle-treated hTau.P301S mice than in vehicle-treated WT mice, but the abundance of *Eva1c* mRNA decreased significantly in NR-treated hTau.P301S mice, while NR did not significantly alter expression of *Eva1c* mRNA in WT mice (Fig. 5B). To systematically assess the impact of NAD^+^ precursors on cellular metabolism, we used both NR and NMN. This sequential approach enabled a direct comparison of their respective contributions to NAD^+^ biosynthesis and downstream biological effects. By analyzing both precursors, we aimed to determine whether the observed cellular responses were precursor specific or a general consequence of NAD^+^ augmentation, thereby providing a more comprehensive understanding of their roles in modulating neuroprotection. A similar decrease in abundance of *EVA1C* mRNA occurred in worm- and cell-based experimental models treated with NAD^+^ precursors NMN or NR (Fig. 5C and table S16). In contrast, EVA1C protein was approximately twofold higher in vehicle-treated control SH-SY5Y cells than in vehicle-treated 2N3R-overexpressing SH-SY5Y cells, and this difference was normalized when the cells were treated with 0.5 mM NMN (Fig. 5, D and E). However, no significant difference in EVA1C protein expression was observed when the cells were treated with 1.0 or 2.0 mM NMN (Fig. 5, D and E). Because changes in *Eva1c* mRNA and EVA1C protein in these two experiments did not concur, the biotype of EVA1C protein was examined (fig. S3, A and B, and table S17). The results show that the changed *Eva1c* transcript in SH-SY5Y cells is a coding sequence, which could explain the discrepancy in experimental results in Fig. 5 (B and C versus D and E).

The roles of EVA1C in neurons and the effects of NR on expression of EVA1C were investigated in hTau[P301L] worms with or without targeted RNAi knockdown of *eva-1*, an ortholog of human *EVA1C*. The results showed improved performance in memory tests in NR-treated versus vehicle-treated hTau[P301L] worms, but *eva-1*–targeted RNAi abrogated this effect (Fig. 5F). The chemotaxis index was used to assess memory ability in worms, with a lower chemotaxis index indicating higher memory capacity and improved performance (*49*). Because previous studies suggest that patients with AD have a shorter lifespan than healthy control groups (*90*), we also investigated the effects of NR and *eva-1* knockdown on the lifespan of hTau[P301L] and WT control worms. Preliminary tests demonstrated that WT control worms had a longer lifespan than hTau[P301L] worms (Fig. 5G), which is in line with published results (*90*, *91*). NR (2 mM) increased the lifespan of hTau[P301L] worms by 16.84% but did not extend the lifespan of *eva-1* knockdown hTau[P301L] worms or *eva-1* knockdown WT control worms (Fig. 5G and table S18), suggesting that *eva-1* is a determinant of lifespan in these worm strains. We further measured pharyngeal pumping in NR-treated and vehicle-treated worms and established that treatment with NR did not change this parameter, a marker of health span (Fig. 5H). In summary, these data argue that NR-induced lifespan extension and the improvement of memory-like capacity are dependent on EVA1C.

### The NAD^+^-EVA1C axis is essential to restore memory deficit in an adeno-associated virus–mediated mouse model of Tau pathology

Encouraged by the nematode data, we next sought to investigate whether NAD^+^-mediated up-regulation of EVA1C could exert similar effects in rodents. To model tauopathy in the mouse brain, we developed a mouse model using adeno-associated virus serotype 8 (AAV8) chicken β-actin (CBA)–blue fluorescent protein (BFP)–2A–Tau–P301S. This system leverages an AAV8 vector, which efficiently transduces neurons in tauopathy mice and enables localized gene expression in the hippocampus (*92*). The construct includes a bicistronic cassette that coexpresses BFP as a fluorescent marker and the Tau protein harboring the pathogenic P301S mutation. A self-cleaving 2A peptide facilitates the independent expression of functional BFP and mutant Tau proteins, enabling precise tracking of transduction efficiency while faithfully modeling Tau-driven neurodegeneration (*93*). This design is consistent with analogous constructs such as AAV8 CBA–yellow fluorescent protein (YFP)–2A–EVA1C and AAV8 CBA-YFP-microRNA (miR)-EVA1C. By using this model, we aim to explore the mechanistic role of EVA1C in mitigating Tau pathology and its broader therapeutic potential.

**Fig. 6.**
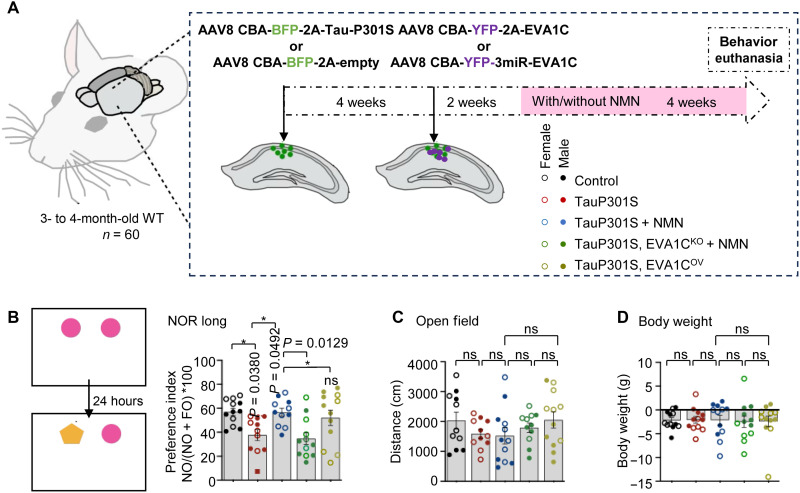
NMN-dependent expression of *Eva1c* improves cognition in tauopathy mice. (**A**) Schematic representation of the experimental design of WT mice injected with AAV8 CBA-BFP-2A-TauP301S or BFP-2A-empty virus for establishing Tau neuropathology. Then, 4 weeks later, the same mice were injected with AAV8 CBA-YFP-2A-EVA1C or 3miR-EVA1C virus for EVA1C overexpression or knocking down, respectively. After 2 weeks, some animals were treated with NMN for 4 weeks; see the five color-coded groups where open circles represent female mice and filled circles indicate male mice. (**B**) Novel object recognition (NOR) was conducted for 2 days, and preference index was calculated in second day (left). hTau.P301S group exhibited reduced preference index in the novel object compared to control group indicating memory deficits. While hTau.P301S + NMN group index was similar to controls (suggestive of memory recovery), this memory recovery was not found in the hTau.P301S + EVA1C^KO^ + NMN group, indicating that the beneficial effect of NMN was lost under EVA1C knockdown conditions (right). The preference index of hTau.P301S, EVAC1^OV^ group was similar to controls supporting the beneficial role of EVAC1 overexpression against Tau-related memory deficits. (**C** and **D**) No difference of total distance traveled in open field (OF) among all groups indicating no changes of locomotion (C), followed by no changes of body weight among all groups (D). Data are means ± SEM. *P < 0.05. Group differences were analyzed with one-way ANOVA followed by Šidák’s multiple comparisons test.

To establish the Tau P301S mouse model, we performed bilateral injections of Tau-P301S-AAV8^OV^ (overexpression) into the hippocampal CA1 region of WT mice. Four weeks postinjection, the same region was further injected with either AAV8 carrying CBA-YFP-2A-EVA1C or CBA-YFP-miR-EVA1C to modulate EVA1C expression. Two weeks after the second injection, designated groups received NMN (denoted as week 6), a NAD^+^ precursor, via drinking water (6 mM, around 7 ml/day) for 4 weeks, followed by behavioral testing (Fig. 6A). The efficiency of AAV injections was evaluated by assessing the fluorescence of BFP, which indicates Tau protein expression, and YFP, which reflects EVA1C expression (fig. S3C).

To evaluate the effects on memory, we conducted novel object recognition (NOR) test. The mice were first exposed to two identical objects for 10 min during the training phase. After 24 hours, one object was replaced with a novel object (Fig. 6B). Exploration time was defined as the duration during which the mouse’s nose was oriented toward the object, and the preference index was calculated accordingly. To assess locomotor activity, we performed the open field (OF) test, where mice were placed in a brightly lit arena and allowed to explore freely for 5 min, while total distance traveled was automatically monitored (Fig. 6C).

**Fig. 7.**
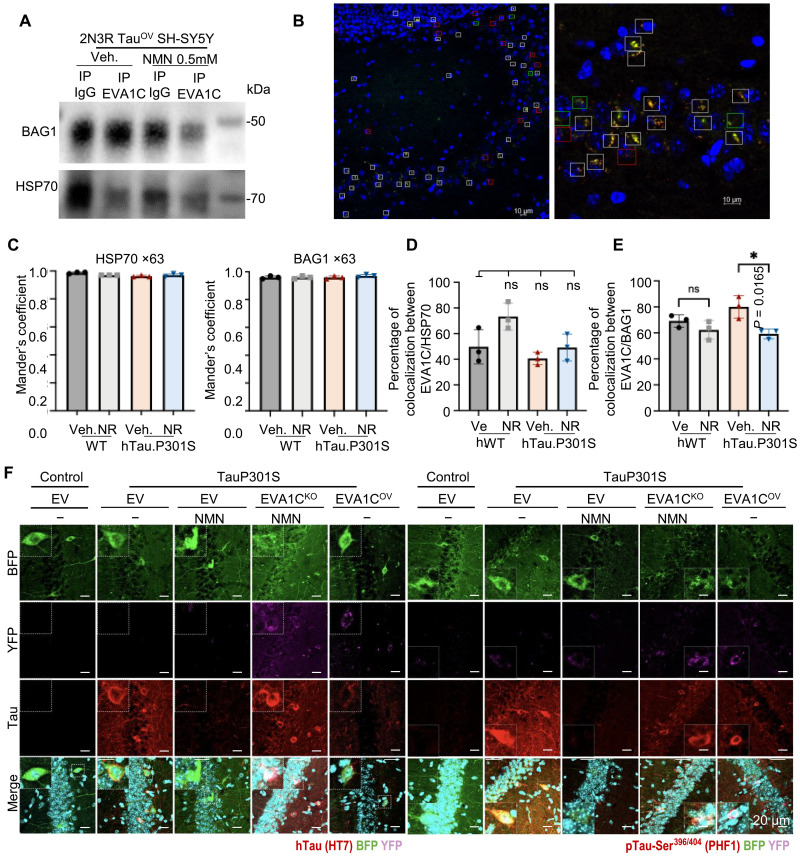
The binding between EVA1C and BAG1 (or HSP70) changes in AD and by NAD^+^ augmentation. (**A**) Co-IP assays investigate the interactions between EVA1C and HSP70 or BAG1. (**B** to **E**) Colocalization analysis between EVA1C and HSP70 or BAG1 in WT and hTau.P301S mice vehicle and treated with NR. (B) Image of hippocampus using WT vehicle mice costained for DAPI (blue), EVA1C (red), and HSP70 (green) at ×20 (left) and ×63 (right). The white squares indicate the presence of colocalization between EVA1C and HSP70, the red squares indicate the presence of only EVA1C, and the green squares indicate only the HSP70 signal. Scale bars, 10 μM. (C) Graphs representing the average of Manders coefficient of the entire image captured at ×63 for all mouse groups (*n* = 3) showing colocalization between EVA1C and HSP70 (left) and between EVA1C and BAG1 (right). [(D) and (E)] Column graphs showing the average of the percentage of number of regions of interest (ROIs) with colocalization between EVA1C and HSP70 (white ROIs) (D) and between EVA1C and BAG1 (E) for all mouse groups (*n* = 3). For mice treated with NR, the colocalization between EVA1C and HSP70 increases, whereas the colocalization between EVA1C and BAG1 decreases. NR significantly decreases the colocalization of EVA1C and BAG1 in hTau.P301S mice. The images were captured at ×20. (**F**) Immunofluorescent analysis of hTau (HT7)–positive cells and PHF1-positive cells in the CA1 hippocampal tissues from the designated mouse groups. EV, empty vector. Data are shown as means ± SEM. *P < 0.05. Statistical significance was assessed by two-way or one-way ANOVA, followed by Šidák’s multiple comparisons test as appropriate. All experiments were performed at least twice. Figure S5 (A and B) shows two sets of co-IP assays related to (A).

In line with the results obtained using *C. elegans* AD-like models, NMN improved recognition memory in the AAV-mediated hTau.P301S mice to the control level as assessed by NOR test, and *Eva1c* overexpression partly mimic the effect of NMN treatment, possibly due to a big variation in the performance of the animals (Fig. 6B). In addition, the benefits from NMN were abolished by *Eva1c* knockdown (Fig. 6B), possibly due to a big variation in the performance of the animals (Fig. 6B), while *Eva1c* knockdown blocked the beneficial effects of NMN treatment in Tauopathy, supporting the mechanistic results presented in Fig. 7F. No changes were observed in total distance (Fig. 6C), indicating no locomotion changes among all groups. These data suggest that the NAD^+^-EVA1C axis protects cholinergic and glutamatergic neurons and preserves memory in worm and mouse models of tauopathy. We further evaluated the impact of NAD^+^-EVA1C axis on body weight; there were no significant differences in body weight between groups at the end point, suggesting no overall effect in animal’s health (Fig. 6D).

### NR promotes the expression of an EVA1C isoform with high affinity for HSP70

We further designed experiments to explore the pathway(s) and effectors downstream of EVA1C in the presence or absence of NR in tauopathy mice. Our approach was to mine genome-wide transcriptomic data for information about differential ASEs and combine the results with predictive modeling of protein 3D structures with AlphaFold 3.

**Fig. 8.**
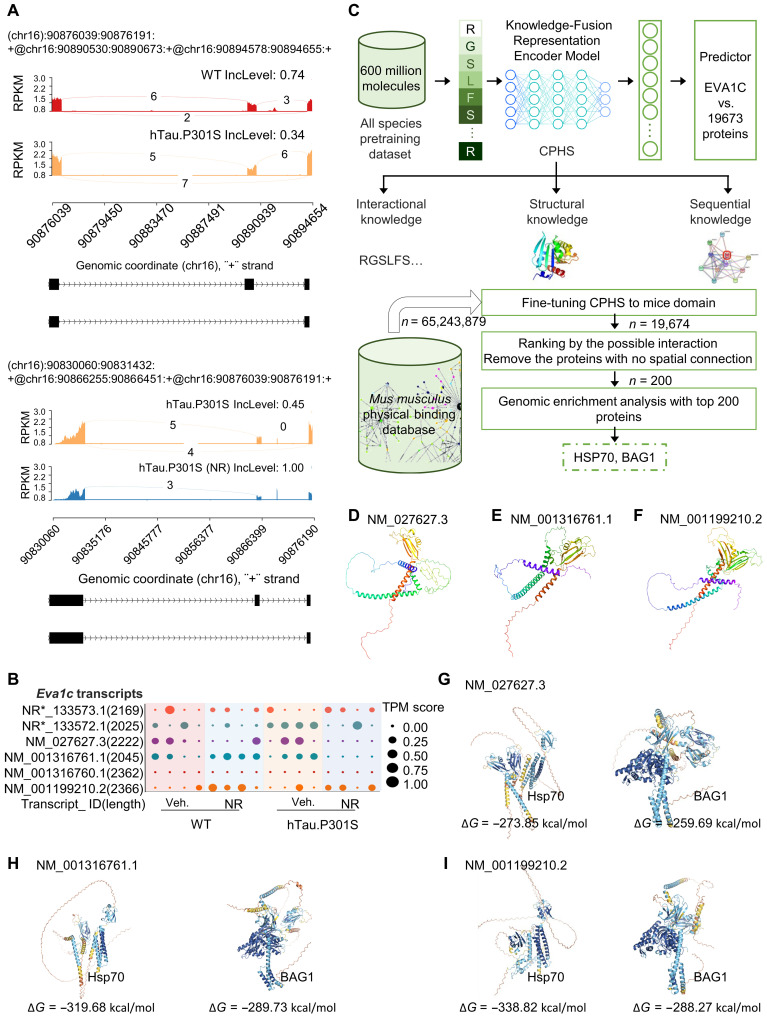
Deep learning strategies to characterize EVA1C PPIs in hTau.P301S and WT mice. (**A**) Sashimi plot of ASEs in *Eva1c* in WT, hTau.P301S, and hTau.P301S + NR mice. Diagrams show the read coverage of exons (top) with IncLevel shown above corresponding diagram. Alternative splicing models are shown schematically below (bottom). The curve indicates the splicing pattern, and the number above the curve is the number of corresponding RNA-seq reads. Similar analysis of an individual sample is in fig. S3D. RNA-seq data from WT, hTau.P301S, and hTauP301S + NR mice are represented in red, orange, and blue, respectively. Reads per kilobase million (RPKM) are plotted against reads split across the splice junction (junction depth) to appear as arcs representing the number of splice junction connected exons per sample. Alternative isoforms are shown below the junction tracks. (**B**) Dot plots representing the relative abundance of six *Eva1c* transcript isoforms under four experimental conditions. (**C**) Schematic outline to illustrate processes of model pretraining and virtual screening. Top: The pretraining dataset was analyzed using the Knowledge-Fusion Representation Encoder Model, and interactional, structural, and sequential knowledge was used to train the EVA1C bonding predictor within the top 0.003%. Bottom: Virtual screening was performed using the *Mus musculus* physical binding database containing 65,243,879 molecules. The pretrained model was fine tuned using mouse domains with 19,674 sequences. The top 100 putative protein interactors were identified and ranked, while proteins with no structural contact were removed from consideration. Genomic enrichment analysis was used to identify two lead hits, HSP70 and BAG1. CPHS, ConfProtein Human Species. (**D** to **F**) Predicted 3D structures of three EVA1C isoforms generated with AlphaFold 3. (**G** to **I**) Protein complexes prediction for EVA1C (green) and HSP70 (gray, purple, and blue) or BAG1(gray, purple, and blue) from AlphaFold 3 and its molecule dynamics simulation results (Δ*G*). The quality assessment of EVA1C protein conformation prediction is presented in fig. S4.

First, ASEs and expression of alternative isoforms of *Eva1c* were quantified in WT control and hTau.P301S mice in the presence or absence of NR (Fig. 8A and fig. S3D). The results reveal differential splicing of the middle exon of *Eva1c*. Specifically, the middle exon of *Eva1c* (90887491:90890939) is differentially included in WT and differentially excluded in hTau.P301S mice (Fig. 8A, top, and fig. S3D). To confirm this result, we analyzed RNA-seq data using the Mixture of Isoforms software. This analysis revealed that the IncLevel (probability of inclusion) of exon 2 was 0.74 versus 0.34 in WT versus hTau.P301S mice, respectively (Fig. 8A, top, and fig. S3D). Furthermore, the right exon (90876190) was differentially excluded in NR-treated hTau.P301S mouse brain (IncLevel of 1.0) and differentially included in vehicle-treated hTau.P301S mice (IncLevel of 0.45) (Fig. 8A, bottom, and fig. S3D). The relative abundance of all *Eva1c* isoforms (*n* = 6) in all experimental groups is summarized in Fig. 8B. Two isoforms are nonprotein-coding transcripts (denoted as “NR*” to distinguish it from the NAD^+^ precursor “NR”), and four are protein-coding transcripts (NM) (Fig. 8B). Noncoding transcripts were significantly down-regulated by NR, suggesting differential expression of active EVA1C in the presence of NR. The protein-coding 2362-bp transcript was not detected in mouse brain (Fig. 8B). Furthermore, expression of the 2366-bp transcript was higher in NR-treated WT and hTau.P301S mice than in the respective vehicle-treated control mice (Fig. 8B); in contrast, NR decreased expression of the 2045- and 2222-bp transcripts in hTau.P301S mice (Fig. 8B). These results could have implications regarding relative expression of functional EVA1C protein in NR- or vehicle-treated tauopathy or WT mice.

Next, we used an AI-based deep learning approach to predicting the EVA1C PPI network and potential downstream effectors of EVA1C in tauopathy models. This was accomplished primarily using AlphaFold to predict EVA1C isoform–specific 3D structures from the EVA1C primary amino acid sequence (Fig. 8, C to F) (*94*). The top five structural models were then identified and used to discover putative PPIs of interest. This analysis focused on three EVA1C isoforms: NM_001316761.1, NM_001199210.2, and NM_027627.3. The top five protein conformations for each isoform were identified after the conformations were ranked on the basis of the local distance difference test (IDDT) (*95*) and the template modeling score (TM score) (*96*). The protein conformations with highest IDDT value and TM score for each isoform were selected for further analysis.

For the three selected protein conformations, >80% of the structure had an IDDT of >0.6, 40% of the structure had an IDDT of >0.75, and all three structures had TM scores of >0.5 (fig. S4). Potential PPIs were evaluated for all three isoforms, and proteins that showed high potential PPIs with all three isoforms were selected for further study. Potential PPIs were fine-tuned using the STRING-mouse species dataset (*97*) as well as DeepPPI (*39*), graph neural network (GNN)–PPI (*98*), PIPR (Protein-Protein Interaction Prediction Based on Siamese Residual RCNN) (*99*), and OntoProtein (*100*) for comparison. Using the STRING-mouse species dataset, *F*_1_ scores were 79.26 ± 0.2 and 87.82 ± 0.4 for the breadth-first search (BFS) and depth-first search (DFS) split training and evaluation datasets, respectively, where higher *F*_1_ score indicates better performance. Our model outperformed DeepPPI, GNN-PPI, and PIPR (table S19).

After fine-tuning the model with the mouse species dataset, PPIs were predicted for the three EVA1C isoforms. After the Ankyrin-repeat, SOCS box protein and the leucine-rich repeat-containing protein were removed because of likely biological irrelevance, HSP70 ranked first in three predictions, and BAG1 ranked in the top 100 (table S20). Because HSP70 is a critically important molecular chaperone and it interacts with BAG1 to induce autophagy (*101*, *102*), subsequent studies focused on interactions between EVA1C, HSP70, and BAG1 (Fig. 8C).

AlphaFold 3 demonstrates robust predictive capabilities in anticipating structures across biomolecular complexes especially protein-protein interfaces. Thus, we used AlphaFold 3 to predict the 3D structure for each isoform with HSP70 and BAG1 complexes. Then, molecular simulations were performed to estimate and compare binding modes and energetics using predicted models of the three isoforms of EVA1C docked with BAG1 or HSP70. The top 10 of 3000 energy-minimized conformations were identified (Fig. 8, G to I). The predicted binding affinities (∆*G*) of HSP70 to NM_001316761.1, NM_001199210.2, and NM_027627.3 isoforms of EVA1C were −319.68, 273.85, and −338.82 kcal/mol, respectively (*P* < 0.05). For BAG1, ∆*G* values for binding NM_027627.3, NM_001316761.1 and NM_001199210.2 were −259.69, −289.73, and −288.27 kcal/mol, respectively (*P* < 0.05). Notably, in hTau.P301S mice, NR promotes differential expression of the NM_001199210.2 isoform of EVA1C, which has higher affinity for HSP70 than for BAG1 (∆*G* = −319.68 versus −289.73). These data indicate significant differences in the binding affinities of EVA1C isoforms with HSP70 and BAG1.

### NR/NMN regulates the interaction between BAG1 and HSP70 and provides benefits in tauopathy via EVA1C

Using SH-SY5Y cells overexpressing 2N3R Tau, the interactions between EVA1C, HSP70, and BAG1 were examined using Western blotting and immunoprecipitation (IP) assays. NR (0.5 mM) slightly up-regulated expression of BAG1 and HSP70 in 2N3R Tau–overexpressing SH-SY5Y cells (fig. S5, A and B). Co-IP assays also indicate that an NR has a more potent impact on the interaction between EVA1C and BAG1 than on the interaction between EVA1C and HSP70 (Fig. 7A and fig. S5B).

To gain deeper insights into the interaction between EVA1C, HSP70, and BAG1 in AD mice, we investigated whether EVA1C and HSP70 (or BAG1) are coexpressed and colocalized in the hippocampal tissue of hTau.P301S mice and normal controls treated with or without NR. Representative immunofluorescence (IF) images illustrate the colocalization of EVA1C (red), 4′,6-diamidino-2-phenylindole (DAPI) (nuclear blue), and HSP70 (green) in hippocampal tissue sections (Fig. 7B). At ×20 magnification (Fig. 7B, left), widespread colocalized EVA1C and HSP70 signals were observed within DAPI-stained nuclei. This finding was further validated at ×63 magnification (Fig. 7B, right), where precise overlapping fluorescence signals confirmed the colocalization of these proteins.

To quantify colocalization, we calculated Mander’s coefficient, a widely used metric ranging from 0 (no colocalization) to 1 (complete colocalization) (*103*). The analysis revealed high colocalization of EVA1C with both HSP70 and BAG1, indicating strong spatial associations between these proteins (Fig. 7C). We also quantified the percentage of regions of interest (ROIs; ~10 μm^2^) displaying colocalized signals (white squares) near neurons. In addition, we determined the percentages of ROIs containing only EVA1C (red squares) or HSP70/BAG1 (green squares) without colocalized signals (Fig. 7, B, D, and E, and fig. S5, C and D). For each mouse, the percentages of colocalized (white), EVA1C-only (red), and HSP70/BAG1-only (green) ROIs were calculated (Fig. 7, B, D, and E, and fig. S5, C and D). Consistent with the cell-based results, IF data showed that NR reduced the colocalization between EVA1C/BAG1 (Fig. 7E) in tauopathy mice but not EVA1C/HSP70 (Fig. 7D).

We further monitored the effects of NMN treatment on common features of Tau pathology in the AAV-mediated hTau.P301S mice by IF staining with hTau (HT7) and pTau-Ser^396/404^ (PHF1) antibodies (Fig. 7F). While NMN treatment reduced hTau (HT7) staining in hippocampal CA1 area of hTau.P301S group, this NMN-driven reduction was blocked in hTau.P301S + *Eva1c*^KD^ + NMN group, indicating that *Eva1c* knockdown attenuated the NMN beneficial effect against the pathological accumulation of hTau; note that these findings are in line with the memory deficits shown in Fig. 6B. Moreover, *Eva1c* overexpression reduced hTau (HT7) staining in hTau.P301S mice (Fig. 7F, left). Next, we found that PHF1 Tau staining was also reduced by NMN treatment, while the NMN effect was partly inhibited when *Eva1c* was knocked down (Fig. 7F, right). Combined, our data show that NAD^+^ augmentation inhibits AAV-mediated Tau pathology in mouse brain via EVA1C.

### EVA1C expression pattern in human tissue–based database and postmortem human brain samples

**Fig. 9.**
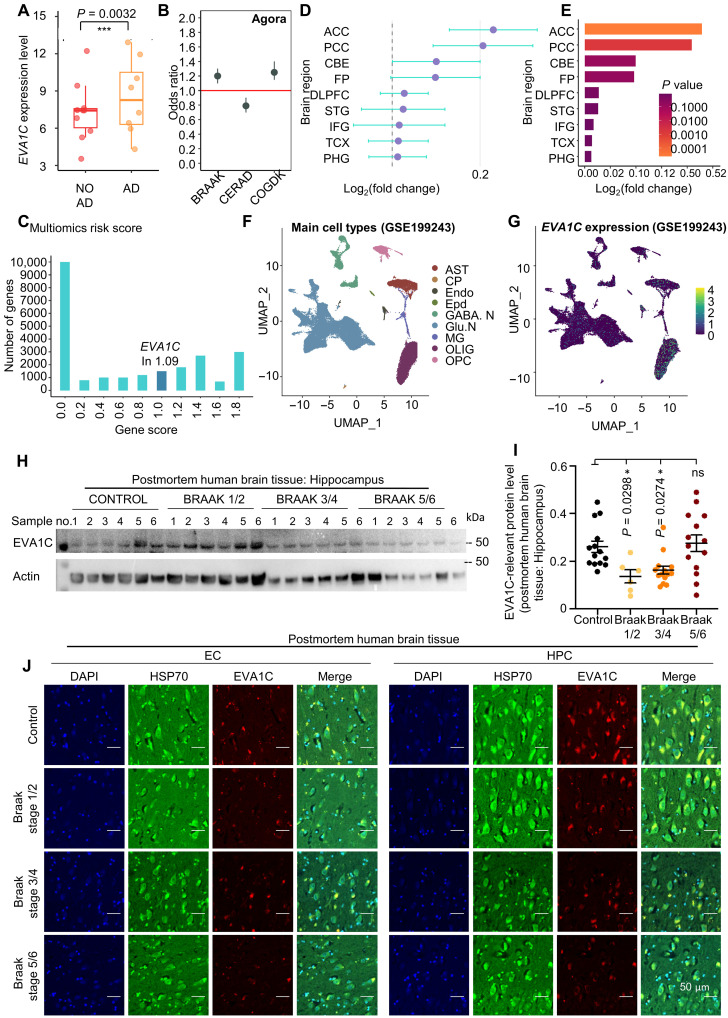
EVA1C expression and analysis in human transcriptomics and brain tissue. (**A**) Box plot showing *EVA1C* differential expression between patients with AD and controls (NO AD) in GSE173955 (*P* = 0.0032). (**B**) Odds ratio analysis of *EVA1C* with AD-related phenotypes. The related transcripts in fig. S5E. (**C**) Multiomics risk score distribution showing gene counts cross score thresholds, with a peak at 1.09. (**D**) Forest plot of EVA1C expression across brain regions. Positive values indicate up-regulation in AD. (**E**) Bar plot of log_2_FC values in *EVA1C* across brain regions, with color intensity reflecting *P* value. (**F**) UMAP (Uniform Manifold Approximation and Projection) of single-cell RNA-seq (GSE199243) showing cell clusters: astrocyte (AST), choroid plexus (CP), endothelial (Endo), ependymal (Epd), γ-aminobutyric acid–releasing neurons (GABA.N), glutamatergic neurons (Glu.N), microglia (MG), oligodendrocyte (OLIG), and OLIG precursor cell (OPC). (**G**) Feature plot of *EVA1C* expression across different cell types in GSE199243, with color gradients 0 to 4. (**H** and **I**) Western blot of EVA1C in hippocampal brain from patients with AD at different Braak stages and age-matched control (6 to 14 samples per group), with quantification (I). Related data are shown in fig. S5F. (**J**) Representative images of immunofluorescent of EVA1C (red), HSP70 (green), and DAPI (blue) in control and Braak stages 1/2, 3/4, and 5/6 (10 samples per group). Representative samples from entorhinal cortex (EC) and hippocampal (HPC) brain regions in fig. S5G. Data are shown as means ± SEM. *P < 0.05 and ***P < 0.001. Statistical significance was assessed by two-way or one-way ANOVA, followed by Šidák’s multiple comparisons test as appropriate. All experiments were performed at least twice.

To answer how EVA1C changes associated with Tau pathologies in the human brain, we performed EVA1C expression pattern analysis via multidimensional analysis in a human-based database and in postmortem human brain tissue. To verify the differential expression analysis, we downloaded and analyzed the RNA-seq dataset GSE173955 from the Gene Expression Omnibus (GEO) database. This dataset contains transcriptomic data from brain tissue samples of patients with AD and non-AD controls (NO AD). The GSE173955 dataset revealed a significant up-regulation of *EVA1C* mRNA in patients with AD compared to controls (NO AD) (*P* = 0.0032) (Fig. 9A). Transcript level analyses (fig. S5E) consistently support our finding in human cells (fig. S3, A and B).

We then used publicly available data from the “Agora” research cohort (*104*), which includes gene expression profiles of patients with AD along with detailed clinical pathology and cognitive function assessments (Fig. 9B). We extracted the expression level of the *EVA1C* gene and calculated its correlation with three clinical indicators: Braak stage, CERAD (Consortium to Establish a Registry for AD) score, and COGDX score. Braak stage and CERAD score reflect the pathological severity of AD (Tau and amyloid plaque, respectively), while COGDX score represents the level of cognitive function. The higher values of Braak staging indicate greater severity of tauopathy; lower CERAD scores indicate greater severity of amyloid plaque; and higher values of COGDX indicate greater severity of cognitive diagnosis. Our data show that *EVA1C* expression was positively correlated with Braak staging and COGDX and negatively correlated with CERAD scores, indicating its close relationship with AD pathology (Fig. 9B). Furthermore, we used multiomics risk scoring data provided by the Agora research cohort. These scores integrate genomic, transcriptomic, and other omics data to evaluate the association between each gene and AD risk. We extracted the risk score of *EVA1C* and analyzed its position within the risk score distribution to assess the potential involvement of *EVA1C* in AD disease risk (Fig. 9C). Multiomics risk score analysis shows a peak at a score threshold of 1.09 including a positive linkage between *EVA1C* and AD.

To evaluate the brain region specificity of *EVA1C* expression, we used gene expression data from multiple brain regions provided by the Agora research cohort. We compared *EVA1C* expression levels across the following nine brain regions: parahippocampal gyrus (PHG), temporal cortex (TCX), superior temporal gyrus (STG), parietal cortex (PC), anterior cingulate cortex (ACC), posterior cingulate cortex (PCC), frontal pole (FP), dorsolateral prefrontal cortex (DLPFC), inferior frontal gyrus (IFG), and cerebellum (CBE). Using the Agora database, we obtained the log_2_(fold change) (log_2_FC) in *EVA1C* expression for each brain region in patients with AD compared to controls (*104*). *EVA1C* expression exhibited distinct increases across brain regions: While PHG and TCX had the most significant changes, IFG and STG also exhibited minor changes (Fig. 9, D and E). These regions are primary sites of AD-related pathological alterations (*105*). To investigate the cell type–specific expression of *EVA1C*, We used the Uniform Manifold Approximation and Projection (UMAP) algorithm for cell visualization and compared the expression levels of *EVA1C* across different neural cell types, including astrocytes (ASTs), choroid plexus (CP), endothelial (Endo), ependymal (Epd), γ-aminobutyric acid–releasing neurons (GABA.N), glutamatergic neurons (Glu.N), microglia (MG), oligodendrocytes (OLIGs), and OLIG precursor cells (OPCs). Particular attention was given to *EVA1C* expression in OLIG (*106*). Single-cell RNA-seq data from GSE199243 reveal distinct expression patterns of *EVA1C* across different neural cell types (Fig. 9, F and G). UMAP visualization highlights a distinct cluster of OLIG with high *EVA1C* expression. OLIGs are critical for myelin formation and maintenance (*107*). Myelin damage and white-matter abnormalities are prominent pathological features in AD (*108*). The high expression of *EVA1C* in OLIGs suggests its potential role in myelin formation, white-matter integrity regulation, and neuronal axon protection. *EVA1C* mRNA is up-regulated in AD, which is in line with the results from AD animal model. Its expression changes are brain region specific, affecting key areas associated with AD pathology. At the cellular level, EVA1C demonstrates a specific expression pattern, particularly in OLIGs, implicating its involvement in myelin and white-matter maintenance.

We then planned to verify our result using postmortem human brain tissue. For this purpose, total lysates were prepared from hippocampal regions of postmortem human brains from patients assigned to different Braak stages or normal control patients. Immunoblotting data show that EVA1C is less abundant in brain tissue from patients at Braak stages 1/2 or 3/4 than in normal controls; samples in Braak stages 5/6 showed high variation (Fig. 9, H and I, and fig. S5F). As the immunoblotting data here were reflections of the average EVA1C protein levels across all brain cell types, we asked whether changes of EVA1C levels occurred specifically in neurons across Braak stages in hippocampus, as well as in the entorhinal cortex [the first region of Tau pathology in AD; see review (*6*)]. We observed around a 30% reduction in number of EVA1C immunoreactive (EVA1C^+^) neurons in the entorhinal cortex of patients at Braak stages 3/4 and 5/6 relative to cognitive normal controls (Fig. 9J and fig. S5G). Similarly, the number of EVA1C^+^ neurons in hippocampal brain tissue was significantly lower in patients at different Braak stages compared to controls (Fig. 9J and fig. S5H). We observed colocalization between EVA1C and HSP70 in neurons (Fig. 8J). Detailed information about patients with AD and their cognitive normal controls is provided (table S21). Collectively, these data suggest that human brain neuronal EVA1C was reduced across all Braak stages in both the entorhinal cortex and hippocampus.

## DISCUSSION

This study demonstrates that NAD^+^-induced differential expression of *Eva1c/EVA1C* isoforms and events downstream of EVA1C contribute to NAD^+^’s neurological benefits in experimental models of AD-like tauopathies. The study uses a multipronged approach involving genome-wide transcriptomic data mining, 3D protein structure modeling using high-accuracy AlphaFold 3 and PPI network predictions, a series of wet laboratory technologies, and different animal models. Computational findings were validated in mouse, worm, and human cell-based tauopathy models, with additional verification using human databases and tissue samples (fig. S6). Our data suggest that differential regulations of ASEs contribute substantially to the therapeutic efficacy of NAD^+^ precursors NR and NMN for treating AD. The results of this study also argue in favor of future development of splice-switching therapeutics for AD and related diseases.

We reported that NAD^+^-regulated ASEs have potential as therapeutic targets for AD, and we also identified genes and ASE-dependent isoforms of these genes that are regulated by NAD^+^ in tauopathy model systems. In this study, transcriptomic analysis of hippocampal mouse brain tissue and a pan-neuronal splicing reporter worm were used to demonstrate dysregulation of ASEs in tauopathy worms. Our result is consistent with results of a recent study on PS19 mice, showing that aberrant ASEs were central contributors to the pathophysiology of tauopathies and represent promising therapeutic targets (*25*). We also demonstrate that neuronal ASEs are dysregulated with age in WT worms in a manner distinct from dysregulation of ASEs associated with pan-neuronal expression of hTau 4R1N; a previous study reported that homeostasis of pre-mRNA splicing serves as both a biomarker and predictor in the gut of aged worms (*46*).

To understand the potential of NAD^+^ in manipulating ASEs for therapeutic purposes, transcriptomic data were mined for information about the biological functions and GO pathways regulated by NAD^+^-stimulated ASEs. We identified pathways associated with axon genesis, axon extension, axon guidance, oxygen metabolism, mitochondrion localization, and autophagy, all of which are primarily induced by NAD^+^ in models of tauopathy. Several previous studies reported that different protein isoforms of the same functional genes have distinct or opposite functions in the neuronal system, including metabotropic glutamate receptor 3, retinal neural cell adhesion molecule, tetratricopeptide repeat–containing Rab8b-interacting protein, quaking homolog (RNA-binding protein), metabotropic glutamate receptor 1, retinoic acid receptor β, excitatory amino acid transporter 2, vascular endothelial growth factor A, brain-derived neurotrophic factor, and plectin (*109*–*116*). Furthermore, the ratios of some mRNA isoforms of these genes were altered by NAD^+^ in tauopathic animals, as observed in the present study. We hypothesize that ASE-regulated genes functionally related to axon development play a critical role in tauopathy and that these genes represent promising targets for NAD^+^-based splice-switching therapeutics for AD.

We found that ASEs in genes involved in RNA splicing are disproportionately dysregulated in tauopathies and aging worms, which is in line with the publications showing that ASE is a mark of aging and neurodegenerative disease (*117*–*120*). This leads us to propose that the beneficial effects of NR in tauopathies may be mediated by its ability to normalize ASEs in these genes. We also found disproportionate NR-dependent effects on A3SS and MES subtypes of ASEs and disproportionate ES events affecting neurons, phenomena that may be important areas of future study. Thus, the prevalence of different ASE subtypes should be considered when designing precision splice-switching therapeutic strategies.

Our transcriptomic data mining identified *EVA1C* splicing as a potentially important target of NR-induced inhibition of tauopathy. Previous studies reported that EVA1C protein was necessary for heparin binding (*121*), which could relate to charge compensation during aggregation of Tau protein (*122*). In addition, a prior study in mice indicated that expression of EVA1C played a role in axon guidance in the murine nervous system (*34*). Here, we found that EVA1C was markedly reduced in the brain tissue of patient with AD, in a pattern crossing Braak stages in both the entorhinal and hippocampal neurons. We also show that NR stimulated expression of EVA1C in an isoform-specific manner and increased EVA1C protein in the brains of hTau.P301S mice.

Computational approaches were used to predict critical PPIs downstream of EVA1C in tauopathy models. This analysis led us to focus on interactions between EVA1C, HSP70, and BAG1, which were subsequently validated in selected experimental models of tauopathy. BAG family proteins are cochaperones of HSP70, and BAG1 can negatively regulate protein refolding by HSP70 in vitro and in vivo (*101*, *123*–*125*). It was proposed that BAG1/BAG3 switching acts as a link between macroautophagy and the ubiquitin-proteasome system (*102*, *126*, *127*). However, little is known about whether and how the BAG1/BAG3 balance influences AD pathophysiology. While BAG1 appears to exacerbate AD pathology and promote neuronal death by stabilizing aggregation-prone Tau species that escape proteasomal degradation (*128*), we envisage that NR may regulate the BAG1/BAG3 balance in an EVA1C isoform–dependent manner in stabilizing of aggregation-prone Tau species; further experiments are necessary. Our further mechanistic studies show that NR/NMN reduced exon skipping events in *Eva1c*; NAD^+^ supplementation increased expression of an EVA1C isoform with higher affinity for HSP70.

Recent studies have identified and characterized PPI-perturbing mutations as key drivers of disease and therapeutic response, advancing precision medicine. For instance, two studies have revealed how disease mutations disrupt PPI, identifying interaction-perturbing mutations linked to cancer progression, patient survival, and drug response, offering important insights for precision medicine (*129*, *130*). These changes could also particularly relevant in neurodegenerative contexts, such as in AD, whereby disease mutation contributes to PPI alterations. Our findings enrich these observations, demonstrating that ASEs markedly affect RNA processing–related PPI networks. Together, these insights underscore a conserved nature (at least partially) of aging-associated PPI network dynamics and highlight the potential of targeting RNA splicing to mitigate age-related functional impairments.

Several limitations exist and unanswered questions warrant further investigation. First, although *Eva1c*/*EVA1C* emerged as a significantly regulated splicing target upon NAD^+^ precursor treatment, we did not examine whether knockdown or knockout of *Eva1c* alone is sufficient to induce abnormal ASEs similar to those observed in tauopathy models. This raises an important mechanistic question of whether *Eva1c* functions as an upstream regulator of splicing homeostasis or acts downstream of other regulatory nodes. Future transcriptomic profiling following *Eva1c* depletion will be essential to clarify its role in ASE dysregulation. Second, while our study establishes a connection between NAD^+^ metabolism and RNA splicing, the precise molecular mediators of this link remain incompletely defined. For example, it remains unclear whether NAD^+^ levels influence splicing via modulation of specific RNA binding proteins (*131*) or through its “partners” such as sirtuins, poly(adenosine 5′-diphosphate–ribose) polymerases, and the RE1-silencing transcription factor (*18*, *65*). Third, our results revealed that *EVA1C* mRNA levels were elevated in AD models, while EVA1C protein levels were reduced, highlighting a disruption of RNA-protein coupling that is consistent with impaired posttranscriptional regulation in neurodegeneration. Differential ASE of *EVA1C* likely contributes to this mismatch, and NAD^+^ supplementation may help restore this imbalance to support protein expression. Further dissection of these pathways, potentially involving NAD^+^-dependent changes in protein acetylation or methylation (*132*), could provide deeper mechanistic insights. Fourth, although our cross-species platform enhances translational robustness, behavioral rescue assays were limited to short-term interventions. Longitudinal studies will be needed to assess the durability of NAD^+^-mediated benefits and to evaluate possible compensatory mechanisms or toxicity arising from chronic NAD^+^ precursor exposure. Fifth, the available bulk RNA-seq database is only representative of gene expression values that are averaged over different conditions. In addition, EVA1C isoform–specific antibodies were not available for use in the present study, which, in turn, ruled out immunologically based approaches to verifying isoform-specific outcomes. To compensate, AI-based deep learning approaches were used to simulate EVA1C isoform–specific interactions and outcomes. Therefore, further investigation is warranted to verify EVA1C isoform–specific interactions with HSP70 and BAG1. Last, while we validated key findings across multiple animal models, confirmations in human-relevant systems such as induced pluripotent stem cell–derived neurons and brain organoids are critical for establishing clinical relevance.

Despite these limitations, the present study provides important insights into NAD^+^-dependent regulation of ASEs in tauopathy and suggests important areas for future development of ASE-targeted therapeutics for AD and AD-like diseases. At the same time, NAD^+^-based therapeutics continue to be relevant for modulating other pathways involved in AD pathogenesis including proteostasis, energy metabolism, immune response, glial function, and neuronal death (*20*, *21*, *133*). Several NAD^+^-related clinical trials targeting AD are in progress (NCT05617508 and NCT04430517), highlighting the importance of further in-depth mechanistic studies. This study contributes to an improved understanding of mechanisms underlying transcriptional diversity, and it could point the way toward successful future development of ASE-targeted precision medicine for treating AD and related dementias.

## MATERIALS AND METHODS

### Bioinformatics and RNA-seq analysis in mice

#### Quality control for raw FASTQ data

Standard next-generation DNA sequencing protocols were used to derive DNA primary sequences from raw data. FASTQ format files were generated containing sequence reads and sequencing quality information. Software packages fastqc and trim_galore were used to remove adapters and N-containing and low-quality reads (<Q20). RNA-seq data from 16 mice were processed and analyzed.

#### Acquisition and alignment of gene-level expression data

Mouse reference genome mm10 and the GTF format gene annotation files were downloaded from the UCSC website (https://hgdownload-test.gi.ucsc.edu/goldenPath/mm10/bigZips/). Then, STAR was used to index the reference genome mm10 and align the clean FASTQ files. Bam files were generated from aligned data using the SAMtools software and were analyzed using RSEM. RSEM was used to calculate and map number of reads and raw counts to corresponding genes. Then, raw counts were corrected for gene length, and influences of sequencing depth and gene length on read count were accounted for. Last, we calculated fragments per kilobase of the exon model per million mapped fragments (FPKM).

#### Analysis of differential expression and gene enrichment at gene level

Pairwise difference comparisons among WT, WT + NR, hTau.P301S, and hTau.P301S + NR (*n* = 4 per group) were performed with the R statistical package DESeq2 (v1.16.1) (*134*). Significance criteria for DEGs were adjusted *P* < 0.05 and absolute log_2_FC > 0.5. Average FPKM values per group were analyzed, gene expression trends were determined, and the fuzzy *c*-means method was used to cluster with normalized values (*135*). GO enrichment analysis and Kyoto Encyclopedia of Genes and Genomes (KEGG) enrichment analysis were performed using R package clusterProfiler (4.0) (*134*). Significance criteria for enrichment of GO and KEGG terms were adjusted *P* < 0.05 (i.e., Benjamini and Hochberg correction).

#### Transcript acquisition and analysis at transcript level

STAR was used to align data from different groups (*136*), and RSEM was used for quantitation (*137*). Count and transcripts per million at the transcript level were analyzed with DESeq2. clusterProfiler and Mfuzz were used to analyze GO and KEGG term enrichment as described above.

#### Acquisition and analysis of ASEs

FASTQ files were analyzed using RSEM, and all five subclasses of ASEs were analyzed using rMATS. The lncLevel value, defined as the percentage spliced in (PSI), represents the fraction of mRNAs corresponding to the inclusion isoform. Absolute values of lncLevel greater than 0.1 and adjusted *P* < 0.05 were considered statistically significant. GO and KEGG enrichment for ASEs per group was also analyzed.

### Bioinformatics and RNA-seq analysis in human

#### Differential expression analysis

We downloaded and analyzed the RNA-seq dataset GSE173955 from the GEO database. This dataset contains transcriptomic data from brain tissue samples of patients with AD and non-AD controls. The method for calculating differential expression follows the procedures described before (*138*).

#### Agora clinical correlation analysis and multirisk scoring

We used publicly available data from the Agora research cohort, which includes gene expression profiles of patients with AD along with detailed clinical pathology and cognitive function assessments. We extracted the expression level of the *EVA1C* gene and calculated its correlation with three clinical indicators: Braak stage, CERAD score, and COGDX score. Braak stage and CERAD score reflect the pathological severity of AD, while COGDX score represents the level of cognitive function.

Furthermore, we used multiomics risk scoring data provided by the Agora research cohort. These scores integrate genomics, transcriptomics, and other omics data to evaluate the association between each gene and AD risk. We extracted the risk score of the *EVA1C* gene and analyzed its position within the risk score distribution to assess the potential involvement of *EVA1C* in AD disease risk. To evaluate the brain region specificity of *EVA1C* expression, we used gene expression data from multiple brain regions provided by the Agora research cohort. We compared *EVA1C* expression levels across the following nine brain regions: PHG, TCX, STG, PC, ACC, PCC, FP, DLPFC, and CER. Using the Agora database, we obtained the log_2_FC in *EVA1C* expression for each brain region in patients with AD compared to controls (*104*).

#### Cell type–specific analysis

We downloaded and analyzed the single-cell RNA-seq dataset GSE199243 from the GEO database, which contains single-cell transcriptomic data from human brain tissue. Preprocessing of the raw single-cell RNA-seq data was performed using the Seurat package (v4.2), including data normalization, dimensionality reduction (PCA), clustering, and cell type annotation. Cell type annotation was based on known cell type–specific marker genes. We used the UMAP algorithm for cell visualization and compared the expression levels of *EVA1C* across different neural cell types, including ASTs, OLIGs, neurons, and MG. Particular attention was given to *EVA1C* expression in OLIGs (*106*).

#### Alternative splicing analysis in humans

We downloaded and analyzed the raw data from the RNA-seq dataset GSE173955 in the GEO database. The methods followed those mentioned above. Briefly, we used the software package rMATS to perform alternative splicing analysis between AD and non-AD groups and visualized the significant ASEs (*P* < 0.05) of *EVA1C* using rmats2sashimiplot.

### AI-based prediction and modeling of protein structures and PPIs

An AI-based deep learning method and pretraining were used to leverage cross-species multilevel structural data and obtain a knowledge-fusion model. The model was refined using mouse data and interaction fields to obtain task-desired representations.

In protein prompt learning, in-context learning leverages extra semantic tokens to steer the model toward task-specific representations. To extend this concept to proteins, we used pretraining task–specific tokens called prompts into the protein sequence, whereby each dedicated sentinel token is associated with a pretraining task and can be leveraged to coax the model toward task-specific protein representations. A prompt-tuning module was used during model refinement. This allowed us to combine prompts on demand and bridge the gap between pretrained knowledge and downstream tasks.

The hierarchical structure–aware model, PromptProtein, was used as a backbone. PromptProtein uses three modules, objective-masked language modeling, α-carbon coordinate prediction, and physical-binding prediction, to acquire primary, secondary, tertiary, and quaternary structural knowledge and the corresponding prompts, including protein sequence [SEQ], protein 3D structure [CRD], and PPI [PPI]. To evaluate this model, we used the PPI benchmark generated by multifaceted PPI prediction based on Siamese residual RCNN | Bioinformatics. The two methods, BFS and DFS, were used to split training and evaluation datasets. The *F*_1_ scores were used as a criterion to compare performance PromptProtein with DeepPPI (*39*), GNN-PPI (*98*), PIPR (*99*), and OntoProtein (*100*) and demonstrate the superiority of our model. Note that the original model is trained on datasets that are not species specific. We first pre–fine tuned parameters of the whole model for mouse using the AlphaFold Protein Structure Database, which contains 79,301 predicted structures, and STRING, which contains 22,048 sequences and 3,126,208 interaction pairs. Initially, the prompt-tuning module was only applied to the STRING dataset. After the initial stage of tuning, the model was used to predict PPI probabilities within the STRING dataset for mouse using EVA1C as the query protein. Nuclear proteins and other proteins intrinsically unlikely to interact with EVA1C were removed, and the top 100 remaining potential interactors were evaluated manually. Detailed methodologies are provided in the Supplementary Materials.

### Protein structure prediction and molecular dynamics

Predicted EVA1C protein structures were generated with AlphaFold 3, which uses an accelerated protein structure prediction algorithm incorporating the fast homology search capability of MMseqs2 with the knowledge base associated with AlphaFold 3 and/or RoseTTAFold. We compared the predicted structure with AlphaFold 3, and there was no significant difference (*P* > 0.05). Then, structures with the highest average pLDDT (predicted local distance difference test) and pTM (predicted template modeling score) score values were used for predicting the PPI.

For predicting the complex and further performing the molecular dynamics simulation for comparison, we applied the AlphaFold 3 server (https://alphafoldserver.com/). The predicted protein-protein complex structures with the highest average pLDDT score are represented in fig. S4, and the PAE (predicted aligned error) score and pTM score are illustrated in fig. S4 and table S12, respectively.

Molecular dynamics simulations used the pdb4amber program to prepare the protein complex. The tags of all noncanonical residues were sorted, and those with the fewest occurrences were merged into the protein structure. The complex files were inspected manually, and the potential of the selected complexes as protein-ligand models was evaluated. We used the AlphaFold 3 server. The server uses a deep learning–based approach to predicting the 3D structures of proteins and protein complexes with high accuracy. The predicted structures with the highest average pLDDT scores, which indicate confidence in the predicted models, were selected and are represented in fig. S4. In addition, the PAE score, which measures the accuracy of the predicted interfaces, and the pTM score, which estimates confidence in the predicted model, are illustrated in Fig. 8 and table S12, respectively.

Using an RTX3080 graphics processing unit, simulations were run over ~30 hours allowing 100 ns per protein-ligand complex with the periodic boundary condition in the NPT ensemble. The detailed steps are as follows: (i) 5000 cycles of minimization (MAXCYC = 5000) in a solvated system using the steepest descent algorithm for cycles 1 to 2500 and the conjugate gradient algorithm for the remaining cycles (MAXCYC − NCYC). (ii) The NVT simulation was heated gradually from 0 to 303.15 K over 500 ps. (iii) The heated system was equilibrated with the NPT ensemble over 1 ns. (iv) Production simulation was run at 303.15 K at ambient atmospheric pressure. The SHAKE algorithm was used to constrain all covalent bonds involving hydrogen; SHAKE does not calculate the forces of hydrogen bonds. (v) Last, a 100-ns production simulation was performed collecting snapshots at 1-ps intervals. MM-PBSA (Molecular Mechanics Poisson-Boltzmann Surface Area) was used to calculate 100 snapshots in the last nanosecond. Conformations with the highest binding affinities are represented graphically in Fig. 8 (G to I). The most probable binding modes were deduced using 100-ns molecular dynamics simulations and MM-PBSA.

Protein interactions play a crucial role in studying protein functions. We apply the “pretrain, fine-tune” paradigm to predict EVA1C isoform–specific interactions. During pretraining, the model learns general protein interaction information. In the fine-tuning phase, it is refined using mouse data. We use a multitask pretraining approach, leveraging information from different structural levels to enhance interaction modeling. We consider three complementary pretraining tasks: (i) masked language modeling (MLM), which captures primary structure information; (ii) coordinate prediction, which acquires secondary and tertiary structure; and (iii) interaction prediction, which acquires quaternary structure.

#### Masked language modeling

This task involves recovering masked amino acid tokens from the available sequence (*139*, *140*). Let Y represent the set of masked tokens and V the vocabulary of amino acids. The MLM loss is defined as follows

$$\begin{array}{c} q\left(y|h_{p}\right)=\frac{\text{exp}\left[p\left(y|h_{p}\right)\right]}{\sum_{v\in Y}\text{exp}\left[p\left(v|h_{p}\right)\right]},\;L_{\text{MLM}}\left(h_{p}\right)=\sum_{y\in Y}-\text{log}q\left(y|h_{p}\right) \end{array}$$ (1)

#### α*-Carbon coordinate prediction*

Secondary structure can be inferred from protein 3D coordinates. We use an α-carbon coordinate prediction task to learn both secondary and tertiary structures. Given the sequence length ∣x∣ , we denote the ground-truth folded 3D structure of the protein as $Z\in {\mathbb{R}}^{|x|\times 3}$ and the structure predictor, a two-layer perceptron network, as κ . The predicted structure is represented as $\kappa \left(h_{p}\right)\in {\mathbb{R}}^{|x|\times 3}$ . By applying translation and rotation to the predicted structure, we minimize the root mean square deviation between the ground truth and predicted structure. The loss is calculated on the basis of this deviation. This approach eliminates the need to handle spatial invariance or equivariance, focusing instead on relative positions between residues. The coordinate prediction loss (CRD loss) is computed as the mean square error (MSE)

$$L_{\text{CRD}}\left(h_{p}\right)=\text{MSE}\left\{Z,\text{Kabsch}\left[к\left(h_{p}\right)\right]\right\}$$ (2)

#### PPI prediction

To capture quaternary structure information, the third pretraining task involves predicting whether two proteins, m th and nth, can interact in a batch. Let $h_{p}^{m}$ represent the m th protein in a minibatch and $y_{m,n}$ be the ground-truth label. We first compute pair-aware protein representation $h_{p}^{m,n}$ and then define the PPI loss as follows

$$\begin{array}{c} \text{Att}n_{m,n}=\text{Sigmoid}\left[\frac{\left(h_{p}^{m}W\right){\left(h_{p}^{n}W\right)}^{T}}{\sqrt{d}}\right]\\ h_{p}^{m,n}=\text{mean}\left(\text{Att}n_{m,n}^{T}\;h_{p}^{m}\right)\;\|\;\text{mean}\left(\text{Att}n_{m,n}\;h_{p}^{n}\right)\\ L_{\text{PPI}}\left(h_{p}\right)=\sum_{m,n\in N}\text{BCE}\left[y_{m,n},p\left(y_{m,n}|h_{p}^{m,n}\right)\right] \end{array}$$ (3)

Here, $W\in {\mathbb{R}}^{d_{W}\times d_{W}}$ is a projection matrix, BCE represents the binary cross-entropy loss function, and N is the batch size.

In multitask training, while tasks can leverage information from one another to enhance performance, interference can also arise because of inconsistent gradient directions (*140*, *141*). To maximize the former and minimize the latter, we use a prompt-guided multitask pretraining and fine-tuning framework. Each pretraining task corresponds to a specific prompt, instantiated as one of three tokens. The task-specific representations are denoted as $h_{\left[\text{MLM}\right]}$ , $h_{\left[\text{CRD}\right]}$ , and $h_{\left[\text{PPI}\right]}$ , respectively. The objective function for prompt-guided multitask pretraining is formulated as follows

$$\begin{array}{c} L=\alpha_{1}L_{\text{MLM}}\left(h_{\left[\text{MLM}\right]}\right)+\alpha_{2}L_{\text{CRD}}\left(h_{\left[\text{CRD}\right]}\right)+\alpha_{2}L_{\text{PPI}}\left(h_{\left[\text{PPI}\right]}\right) \end{array}$$ (4)

During multitask pretraining (as shown in Eq. 4), both the model parameters ψ and prompts p are optimized. This allows the model to learn task-specific optimal representations rather than a single representation for all tasks, thereby reducing task interference.

In addition, to bridge the gap between pretraining and downstream tasks, the model can flexibly combine learned prompt tokens through prompt tuning, allowing it to mix the acquired information as needed. We define the prompt-tuning module as $\tau_{\theta }\left(\cdot \right)$ . The desired protein representation for downstream tasks, $h_{p\prime }$ , is obtained by feeding the tuned prompt $p^{\prime }$.

$$p^{\prime }=T_{\theta }\left(p_{\left[\mathrm{MLM}\right]},p_{\left[\mathrm{CRD}\right]},p_{\left[\mathrm{PPI}\right]}\right)$$ (5)

The pretrained model then generates $h_{p\prime }$ , which can be used to make predictions for the downstream task. Equation 5 demonstrates how pretraining task information can be flexibly used during the fine-tuning stage. During pretraining, only one prompt is appended to acquire task-specific information. However, in the fine-tuning stage, all learned prompt tokens are fed into $\tau_{\theta }\left(\cdot \right)$ allowing flexible combinations of the acquired information. In this approach, we use a linear layer as the prompt-tuning module to combine the three learned prompts.

### Human brain samples

#### Ethical

The study was performed in accordance with the Helsinki Declaration and Principles for Ethical Research. All participants gave informed consent in writing. The regional Ethics Committee for Medical Research in the South-East of Norway (REK 82685) and the local Data Protection Officer approved the study.

#### Western blot

Western blot assays were done as previously described (*60*). Briefly, human or mouse brain samples were collected and homogenized in tissue homogenizer (Next Advance) with 0.5-mm zirconium oxide beads and ice-cold Triton X-100 buffer containing 1× protease inhibitor (catalog no. B14002, Bimake) and 1× phosphatase inhibitor (catalog no. B15002, Bimake). For homogenization, the tube was shaken at 4°C at maximum speed for 5 min and then centrifuged (21,000*g*) for 10 min at 4°C. The supernatant was then collected as a soluble fraction. The pellet was washed three times with 1% Triton X-100 buffer, and the insoluble pellet was solubilized with 1× radioimmunoprecipitation buffer containing 8 M urea, 5% SDS, 1× protease inhibitor, and phosphatase inhibitor. The sample was then centrifuged (21,000*g*) for 10 min at 4°C, and the supernatant was collected as insoluble fraction. The protein concentration was tested via the bovine serum albumin (BSA) method: The insoluble fraction of protein sample (30 μg) was prepared and denatured at 95°C for 10 min and used in this study. NuPAGE 4 to 12% bis-tris protein gel (catalog no. NP0336BOX, Thermo Fisher Scientific) was used to separate proteins run at 200 V for 45 min. The transfer system was set at 25 V for 30 min on polyvinylidene difluoride membrane (for all the proteins from 15 to 350 kDa). Various antibodies were probed. BSA (5%) was dissolved in tris-HCl buffer containing 0.1% Tween 20 (1× TBST) and used to block nonspecific binding. Blots were incubated with Eva1 homolog C antibody (1:500) (catalog no. NBP1-88937, Novus), and actin (1:5000) (MA1-744, Invitrogen) at 4°C overnight, respectively. Blots were washed three times with 1× TBST and incubated with either anti-rabbit immunoglobulin G (IgG) horseradish peroxidase (HRP)–linked antibody (7074, Cell Signaling Technology) or anti-mouse IgG HRP-linked antibody (7076, Cell Signaling Technology) with a dilution of 1:5000 at room temperature for 1 hour. ChemiDoc XRS System (Bio-Rad Laboratories) was used to detect chemiluminescence. Quantification was performed using ImageJ.

#### IF/immunohistochemistry

Human brain sections were prepared from the prefrontal cortex, entorhinal cortex, and hippocampus of patients at designated Braak stages 1/2, 3/4, or 5/6 and normal healthy controls. Only one brain region/Braak stage was sampled per individual patient/control subject. Tissue sections (7 μM) were slide mounted and then sequentially dehydrated in xylene, 50% ethanol, and H_2_O_2_. Subsequently, sections were rinsed 3× for 10 min in PB [125 nM phosphate buffer (pH 7.4)]. After preincubation in 5% BSA TBS-TX (50 mM tris, 0.87% sodium chloride, and 0.5% Triton X-100) for 1.5 hours, the sections were incubated with primary antibodies (1:300 in TBS-TX) at 4°C overnight. After rinsing, sections were incubated with secondary antibody (1:1000) and washed 3× for 10 min in PB. Slides were covered with ProLong gold antifade with DAPI reagent (P36931, Invitrogen) and imaged using an automated slide scanner/fluorescent imaging system (Axio Scan Z1, Zeiss). Digital images were captured with a 20× objective (numerical aperture, 0.8). ZEN lite Blue software was used for data analysis, and image quantification used a 5-μm 2 × 2 square grid equidistantly distributed over the brain ROI. In each section and region, eight sampling areas were selected randomly and quantified. The EVA1C/BAG1 and EVA1C/HSP70 colocalization index was quantified in a similar manner.

### Cell culture for mechanistic studies

Human neuroblastoma SH-SY5Y and 2N3R Tau–overexpressing SH-SY5Y cells (generated by M. Akbari) were used in this study. The cells were maintained in Gibco Dulbecco’s modified Eagle’s medium (DMEM) supplemented with 10% fetal bovine serum (FBS) with 1% penicillin-streptomycin at 37°C under 5% CO_2_. After treatment, cells were collected for Western blot or co-IP assay.

The co-IP assay was performed as previously described (*60*). Subsequent protein analysis via Western blot was carried out as described above using Eva1 homolog C antibody (catalog no. NBP1-88937) (1:1000 dilution, primary). Secondary antibody was diluted 1:5000 in blocking buffer.

#### Co-IP assays

SH-SY5Y cells were trypsinized, washed twice with ice-cold phosphate-buffered saline (PBS), released, and collected by centrifugation. Cell pellets were flash frozen in liquid N_2_, then resuspended in 2-PCV (packed cell volume) hypotonic lysis buffer [20 mM Hepes (pH 7.9), 2 mM MgCl, 0.2 mM EGTA, 10% glycerol, 0.1 mM phenylmethylsulfonyl fluoride (PMSF), 2 mM dithiothreitol (DTT), 1× Halt protease inhibitor cocktail], placed in liquid nitrogen for 5 min, and then subjected to three freeze/thaw cycles. After the last freeze/thaw cycle, NaCl and NP-40 were added to final concentrations of 0.5 M and 0.5% (v/v), respectively, and then incubated for 20 min on ice. The samples were diluted with 8-PCV hypotonic lysis buffer containing 50 mM NaCl and sonicated. Nucleic acids were degraded by adding either 1.2 μl of CaCl_2_ (2.5 M) and 25 U of deoxyribonuclease or 2.1 μg of ribonuclease I and 50 U of micrococcal nuclease. Nuclease reactions were incubated for 1 hour at 4°C. Samples were centrifuged, supernatants were collected, and aliquots of the supernatant (0.5 mg) were incubated with 2 μg of anti-IgG antibody, anti-EVA1C (catalog no. NBP1-88937, Novu), or IgG [rabbit IgG (C15410206, Diagenode) or mouse IgG (C15400001, Diagenode)] at 4°C overnight. Protein A Dynabeads or Protein A sepharose beads were equilibrated in IP buffer at 4°C overnight, washed three times, added to samples, and incubated for 4 hours at 4°C. The supernatant was removed, and beads were washed three times with cold wash buffer. Beads were boiled in Laemmli buffer and analyzed by Western blot as described.

#### C. elegans

##### C. elegans drug treatment and RNAi experiments

*C. elegans* were cultured at 20°C on standard nematode growth medium (NGM) agar plates with OP50, unless stated otherwise. NR was custom synthesized by Maintain Biotech Co. Ltd. (1094-61-7) and prepared at 1, 2, or 5 mM in sterile distilled water. Worms were treated with drugs from day 1 (day of hatching); RNAi was delivered to worms on treatment plates. *C. elegans* strains and genetics used in this study are listed below:

1) *ybEx2566 [rgef-1::C33H5.18E1E2(+1)E3-P2A-mCherry + rgef-1::C33H5.18E1E2(+1)E3-(+2)P2A-EGFP + pBSKII(−)]* (KH2566, Kuroyanagi Hidehito Lab)

2) *sid-1(pk3321) V;uIs69 [pCFJ90 (myo-2p::mCherry) + unc-119p::sid-1] V;ybEx2566 [rgef-1::C33H5.18E1E2(+1)E3-P2A-mCherry + rgef-1::C33H5.18E1E2(+1)E3-(+2)P2A-EGFP + pBSKII(−)]* (EFF084, The Fang Lab)

3) *Is[Paex-3::tau4R1N(P301L)*, *Pmyo-2::gfp]; ybEx2566 [rgef-1::C33H5.18E1E2(+1)E3-P2A-mCherry + rgef-1::C33H5.18E1E2(+1)E3-(+2)P2A-EGFP + pBSKII(−)]* (EFF084, The Fang Lab)

4) *lin-15 (n765) ybIs2167 [eef-1A.1::RET-1E4E5(+1)E6-GGS6-mCherry eef-1A.1::RET-1E4E5(+1)E6-(+2)GGS6-EGFP lin-15 (+) pRG5271Neo] X* (KH2235, Kuroyanagi Hidehito Lab)

5) *Is[Paex-3::tau4R1N(P301L)*, *Pmyo-2::gfp];ybEx2261 [Peef-1A.1::C33H5.18E1E2(+1)E3-GGS6-mCherry*, *Peef-1A.1::C33H5.18E1E2(+1)E3-(+2)GGS6-EGFP pBSKII(−)]* (EFF023, The Fang Lab)

6) *sid-1(pk3321) V;uIs69 [pCFJ90 (myo-2p::mCherry) + unc-119p::sid-1] V* (TU3401, The Rafal Lab)

7) *Is[paex-3::tau4R1N(P301L) + pmyo-2::gfp];sid-1(pk3321) V;uIs69 [pCFJ90 (myo-2p::mCherry) + unc-119p::sid-1] V* (EFF029, The Fang Lab).

#### Short-term memory assay

Chemotaxis toward an attractant [i.e., isoamyl alcohol (IA)] on a 10-cm agar plate was performed at 20°C, as previously described (*60*). More specifically, 200 to 300 synchronized day 1 worms were collected, washed five times with M9 buffer, and placed on 6-cm NGM plates (without OP50) with IA for 120 min conditioning or without IA for 120 min. For preconditioning, 10 μl of pure IA was placed in the middle of the plate lid at the start of the incubation period. Assay plates were prepared 30 min before starting the assay by adding 20 μl of 20 mM NaN_3_, covering the “IA” treatment area with a 0.5 cm–by–0.5 cm piece of Parafilm. Then, worms were collected in M9 buffer, quickly dried with tissue paper, and placed on the “source point,” and 4 μl of diluted IA (1:50) was added to the Parafilm. The testing plates were then quickly sealed with Parafilm. After 120 min, the number of worms in the vicinity of source points S, IA, and “T” was counted. The worm memory–like behavior was calculated using the chemotaxis index, which was calculated as (#IA − #T)/(#IA + #T + #S), where “#” denotes numbers, IA denotes “gradient” region, T denotes “trap” region, and S denotes start point. For detailed methodology, see our laboratory’s previous publication (*142*). A lower test score correlates with higher memory capacity/performance. Three to five biological replicates were performed.

### Lifespan and pharyngeal pumping rate assays

#### Lifespan assay

Lifespan assays were conducted at 20°C on 3.5-cm NGM plates as described previously (*60*). Drugs were diluted in water (i.e., 2 mM NR) and added in plates before experiments. Synchronous animals were generated via bleaching under defined conditions. Thirty-five to 40 L4 larvae per technical repeat were transferred to 3.5-cm plates. In one biological repeat, at least 100 synchronous worms from three technical repeats were tested. The plates were changed twice a week to ensure continuous exposure to the same drug dosage. Worms were scored daily, and the number of living and dead worms was recorded. Criteria for death were absence of pharyngeal pumping and unresponsiveness to touch. Worms that died because of gonad extrusion, internal bagging, or crawling on the edge of the plates were censored and given a weight of “0,” so they did not contribute to mortality calculations. The log-rank test (Mantel-Cox) was used to calculate the mean, the SD of the mean, and the *P* value. The Kaplan-Meier analysis was used to derive survival curves. Statistical analyses were carried out using Prism (GraphPad Software).

#### Pharyngeal pumping rate

Animals were synchronized and maintained as described for the lifespan assay. The number of pharyngeal contractions in 30 s per adult worm was counted manually (*60*).

### Production of AAV8 CBA-EYFP-T2A-mEVA1C, AAV8 CBA-EYFP-miRNA mEVA1C, and AAV8 CBA-EGFP-2A-TauP301S

A plasmid construct, pAAV CBA–enhanced YFP (EYFP)–T2A–mEVA1C, was generated to drive the expression of mouse mEva1c(NM_001199210.2) with EYFP at the N terminus, under the control of the ubiquitous cytomegalovirus early enhancer fused to CBA promoter. The pAAV construct for AAV8 CBA-EYFP-miRNA mEVA1C was designed and engineered to coexpress EYFP along with three miRNAs targeting different regions of mEva1c. For the AAV8 CBA–enhanced GFP (EGFP)–2A–TauP301S construct, the amino acid leucine at position 301 in the pAAV CBA-EGFP-2A-TauP301L was mutated to serine, and the EGFP was replaced with mTagBFP.

The constructs were packaged in AAV8 and purified using the iodixanol density gradient method described below. Specifically, the transgene containing construct was transfected into the AAV-293 cell line (CVCL_6871, Agilent, USA) along with AAV helper plasmids encoding the necessary structural elements. The day before transfection, 8 × 10^6^ AAV-293 cells were seeded into 150-mm cell culture plates in DMEM containing 10% FBS (catalog no. 16000-044, Thermo Fisher Scientific, USA) and penicillin-streptomycin. Polyethylenimine-mediated cotransfection of the pAAV plasmid containing the transgene, pHelper, and pAAV8 capsid helper plasmids was performed the next day. After 24 hours, the medium was replaced with fresh DMEM containing 10% FBS. The AAV-293 cells were cultured in a 37°C and 5% CO_2_ incubator for 2 days posttransfection to allow recombinant AAV (rAAV) synthesis. The medium and cells containing virus particles were scraped from the culture plates and isolated by centrifugation at 250*g*. The cell pellet was lysed using a buffer containing 10 mM tris, 150 mM NaCl, and 10 mM MgCl_2_ (pH 7.6). The supernatant was mixed with 40% polyethylene glycol (PEG) and incubated on ice for 2 to 12 hours to precipitate virus particles. The PEG-treated medium was centrifuged at 4000*g* for 15 min. The lysate and PEG precipitate were treated with Benzonase nuclease HC (catalog no. 71206-3, Millipore) for 45 min at 37°C. Benzonase-treated lysate was centrifuged at 3000*g* for 15 min, and the clear supernatant was subjected to iodixanol gradient ultracentrifugation. A gradient consisting of 15, 25, 40, and 58% iodixanol was prepared in a Beckman quick-seal centrifuge tube, with phenol red added to the 25 and 58% layers for visualization. The virus-containing supernatant was carefully layered above the 15% iodixanol. The tube was sealed using a heating device (Beckman and Coulter). The 40% iodixanol layer was collected after ultracentrifugation (350,000*g* for 90 min at 10°C) and buffer exchanged with PBS using Amicon ultra centrifugal filters (catalog no. Z648043, Millipore), following a modified protocol from Addgene, USA. Quantitative polymerase chain reaction was performed on the viral stock, determining the titer to be ~10^13^ infectious particles/ml.

### Transgenic mice and AAV-mediated mouse model

#### Transgenic mice

Thy1-hTau.P301S transgenic mouse procedures and protocols were approved by the Jinan University Institutional Animal Care and Use Committee. The Thy1-hTau.P301S mouse strain was a gift from M. Goedert (Cambridge, UK). The mouse strain was constructed as described previously (*143*). The animal experiments conducted in Norway were approved by the regional Ethics Committee for Medical Research in South-East Norway (FOTS ID 16060).

#### AAV-mediated mouse model

WT mice with C57BL/6J genetic background (3 to 4 months old, males and females distributed equally among experimental groups) were used in this study. All animals were housed under standard laboratory conditions, including a controlled 12-hour light/dark cycle (lights on from 08:00 to 20:00), an ambient temperature of 21° ± 1°C, and a relative humidity of 50 to 60%. They had unrestricted access to food and water. Health monitoring followed the Federation of European Laboratory Animal Science Associations guidelines, confirming the specific pathogen–free status of sentinel animals kept in the same room. All procedures complied with European regulations (EU Directive 2010/63/UE). The animal facilities and personnel involved in experiments were certified by the Portuguese regulatory authority, Direcção Geral de Autoridade Veterinária.

For virus information, all viral tools used in this study were provided and packaged in Kavli Institute for Neuroscience, NTNU (Norwegian University of Science and Technology). The AAVs were packaged into eight serotypes with final titers at least 2 × 10^12^ genome copies/ml as described above. AAV8 CBA-BFP-2A-Tau-P301S was injected to 2- to 4-month-old mice to create AAV-mediated mouse model. After 4 weeks, AAV8 CBA-YFP-2A-EVA1C or AAV8 CBA-YFP-3miR-EVA1C was injected.

Before surgery, mice were administered Bupaq (1 μl/mg, intraperitoneally). Anesthesia was induced in a gas chamber using sevoflurane and confirmed by the absence of withdrawal responses to hindlimb pinching. Injections were performed bilaterally in the CA1 hippocampal region using a Hamilton syringe, with stereotaxic coordinates (relative to Bregma) set at anteroposterior of −1.9, mediolateral of ±1.4, and dorsoventral of −1.5, according to Paxinos and Franklin (*144*). A dental drill was used to perform a craniotomy (∼0.5 mm in diameter) above the target areas. Two microliters was microinjected at an infusion rate of 0.25 μl/min, followed by 7 min of rest before removal of the needle to prevent withdraw of the solution. The first virus injection delivered AAV8-CBA-BFP-2A-Tau P301S or AAV8-CBA-BFP-2A to all animals, while the second virus injection introduced (see below and Fig. 8A) either AAV8-CBA-YFP-2A-EVA1C or AAV8 CBA-YFP-3miR-EVA1C in specific animal groups; the remaining animals were subjected to the complete anesthesia protocol and stereotaxic protocol.

NMN (MT1250221B, Maintain Biotech Co. Ltd.) treatment began 2 weeks after the second injection with 6 mM, around 7 ml/day in drinking water for 4 weeks, as previous described (*21*), and continued until the animals were euthanized. Control groups received drinking water only. To minimize potential variability from compound degradation, NMN-containing drinking water was replaced twice weekly. NMN concentration was monitored throughout the experimental period and remained stable at >95%. Water consumption and body weight were monitored throughout the experimental timeline. Animals were maintained and fed under standard conditions during the entire experiment. The experimental groups included control (empty-AAV), TauP301S (TauP301S-AAV), TauP301S + NMN (TauP301S-AAV and NMN), TauP301S + EVA1C^KO^ + NMN (TauP301S-AAV and TauP301S + EVA1C^ov^ and NMN treatment), and TauP301S + EVA1C^ov^ (TauP301S-AAV + EVA1C^ov^) groups, using 11 to 12 animals per group and gender balanced. See schematic representation of experimental design and timeline in Fig. 8A.

#### Behavior testing in AAV-mediated mouse model

The NOR test was conducted to assess recognition memory. The test was carried out in a square arena (33 cm by 33 cm by 33 cm) with white walls and floor. Mice were habituated to the empty arena for 20 min per day over 3 consecutive days. On the fourth day, mice were exposed to a pair of identical objects for 10 min. After 24 hours, one of the objects was replaced with a novel object. All sessions were videotaped and scored manually using Kinoscope software (https://sourceforge.net/projects/kinoscope/). Exploration time was defined as the time the mouse’s nose was facing the object and the preference index was calculated.

The OF test was conducted to evaluate locomotor activity in mice. The apparatus consisted of a brightly illuminated square arena with white walls and floor (46 cm by 46 cm by 33 cm). Each mouse was gently placed in the center of the arena and allowed to explore freely for 5 min. The arena was cleaned with a 10% ethanol solution between trials to prevent odor interference. Each trial was recorded with a camera, and the analysis was performed using the Y-maze software (Stoelting Co., Wood Dale, IL, USA). The total distance traveled served as a measure of locomotor activity.

#### Hippocampal sample acquisition and mRNA extraction and detection from transgenic mice

Hippocampal tissue from Thy1-hTau.P301S mice was resuspended in TRIzol and dispersed by shaking. Aliquots were separated by agarose gel electrophoresis to ascertain RNA integrity and purity. NanoPhotometer spectrophotometry and the Agilent 2100 Bioanalyzer were also used to confirm purity and integrity of RNA samples, respectively.

#### Library construction and quality control for the transgenic mice

Extracted mRNA (total ≥ 800 ng) was used to build the library with Illumina’s NEBNext Ultra RNA Library Prep Kit. The oligo(dT) magnetic beads were used to enrich for polyadenylate-containing mRNA as input for library construction. To ensure the quality of the library, the effective concentration (no less than 2 nM) was accurately quantified. DNA sequencing was performed according to Illumina DNA sequencing protocols.

#### Colocalization analysis in transgenic mice

Brain tissue samples from each group of mice, WT-vehicle, WT-NR, Thy1-hTau.P301S-vehicle, and Thy1-hTau.P301S-NR, were immunostained for DAPI and antibodies for EVA1C and HSP70. Another series of tissue samples from the same groups of mice were immunostained with DAPI and antibodies against EVA1C and BAG1. The signals from DAPI and EVA1C were blue and red, respectively, whereas the signals from HSP70 and BAG1 were both green.

To quantify the colocalization between EVA1C/HSP70 and EVA1C/BAG1, we performed colocalization analysis using both ×20 and ×63 images via the “Coloc” tool in the ZEN 2.3 software (Carl Zeiss). First, we quantified the number of colocalized pixels in the whole image using the Manders coefficients, which is an indicator of the overlapping signals representing the degree of colocalization where the values range from 0 to 1 (where 1 represents perfect colocalization between the two channels) (*103*, *145*). Afterward, we quantified the percentage of number ROI of about 10 μm^2^ that showed colocalized pixels (white square) in the proximity of neurons. We also quantified the percentage of ROIs that showed only EVA1C (red square) and HSP70 or BAG1 (red square) signals, without colocalized ROIs. To calculate the percentage of ROIs, the ROIs were drawn with ZEN software including the pixels of interest. The ROI colors were drawn according to the pixels’ color (e.g., a white square was drawn around a white pixel, indicating colocalization). Afterward, the total number of ROIs related to the colocalization between EVA1C and HSP70 or BAG1 (white), to only EVA1C (red), and only HSP 70 or BAG1 (green) was annotated for each mouse. For each mouse, the percentage of colocalization between EVA1C and HSP70 or BAG1 (white ROIs), percentage of EVA1C (red ROIs), and percentage of HSP70 or BAG1 (green ROIs) was calculated. The percentage was calculated dividing the number of white (colocalization), red (EVA1C), or green (HSP70 or BAG1) ROIs by the total number of ROIs (white + red + green) of the entire image and multiplying the result by 100 (Fig. 7, B to E, and fig. S5, C and D). The percentages were calculated using Microsoft Excel 2016 (Microsoft Corporation, 2016).

#### Brain analysis in AAV mediated-mouse model

For euthanasia, mice were deeply anesthetized [ketamine hydrochloride (150 mg/kg) plus medetomidine (0.3 mg/kg)] and transcardially perfused first with saline and then with 4% paraformaldehyde. Brains were then collected and postfixed in 4% paraformaldehyde for 6 hours and transferred to a 30% (w/v) sucrose solution until they sank. After impregnation with sucrose, the brains were immersed in a 2% (w/v) agarose solution and sectioned coronally in slices with a thickness of 30 μm, using a vibratome (VT1000S, Leica, Germany). Brain slices were maintained in PBS solution at 4°C until the IF staining protocol.

The IF staining protocol was the same for all the primary antibody used: HT7 (1:500; #MN1000, Invitrogen). For HT7 the secondary antibody used was donkey anti-mouse Alexa Fluor 594 (1:1000; #A-21203, Invitrogen). Briefly, brain slices were washed twice with PBS, and antigen retrieval was performed [10 mM citrate buffer (pH 6.0), Sigma-Aldrich; 20 min at 80°C], followed by permeabilization with 0.5% (v/v) Triton X-100 (Sigma-Aldrich, USA) in PBS solution for 20 min. The slices were then incubated in a blocking solution containing 5% (v/v) FBS in 0.3% (v/v) PBS–Triton X-100 for 30 min at room temperature, washed and incubated with the primary antibody, and diluted in 0.3% (v/v) PBS–Triton X-100 with 1% (v/v) FBS overnight at 4°C. Secondary antibody incubation was followed by 10-min incubation with DAPI (1:1000; #D1306, Invitrogen) at room temperature. Slices were washed and mounted using an aqueous mounting medium (Epredia Lab Vision PermaFluor Aqueous Mounting Medium). Images were collected by confocal microscopy using Olympus FLUOVIEW FV3000 and processed with Fiji software.

### Statistical analysis

All “wet lab” data are presented as means ± SEM, unless otherwise specified. A two-tailed unpaired *t* test was used for pairwise comparisons between groups. Group differences were analyzed using one-way analysis of variance (ANOVA) followed by Šidák’s multiple comparisons test or two-way ANOVA followed by Tukey’s or Dunnett’s multiple comparisons test for multiple groups and determined to be normal distributions. For other datasets, Mann-Whitney *U* or Kruskal-Wallis tests were used. All statistical analyses used GraphPad Prism 8.0 software. The criterion for statistical significance was *P* < 0.05.

## Supplementary Materials

### The PDF file includes:

Figs. S1 to S6 Tables S4, S18, S19, and S21 Legends for tables S1 to S3, S5 to S17, S20, S22, and S23 Western blot full scans
