# Supplementary material for: NAD+ reverses Alzheimer’s neurological deficits via regulating differential alternative RNA splicing of EVA1C
Source: Sci Adv. 2025 Nov 7;11(45):eady9811. doi: 10.1126/sciadv.ady9811 (PMC12594206; doi:10.1126/sciadv.ady9811)
Supplement: Supplementary file 1 — Figs. S1 to S6 Tables S4, S18, S19, and S21 Legends for tables S1 to S3, S5 to S17, S20, S22, and S23 Western blot full scans [file sciadv.ady9811_sm.pdf]

Science Advances
AAAS

Supplementary Materials for

NAD$^+$ reverses Alzheimer's neurological deficits via regulating differential alternative RNA splicing of *EVA1C*

Ruixue Ai *et al.*

Corresponding author: Joana Margarida Silva, joanamsilva@med.uminho.pt; Oscar Junhong Luo, luojh@jnu.edu.cn; Evandro Fei Fang, e.f.fang@medisin.uio.no

Sci. Adv. **11**, eady9811 (2025)
DOI: 10.1126/sciadv.ady9811

The PDF file includes:

Figs. S1 to S6
Tables S4, S18, S19 and S21
Legends for tables S1 to S3, S5 to S17, S20, S22 and S23
Western blot full scans

Other Supplementary Material for this manuscript includes the following:

Tables S1 to S3, S5 to S17, S20, S22 and S23

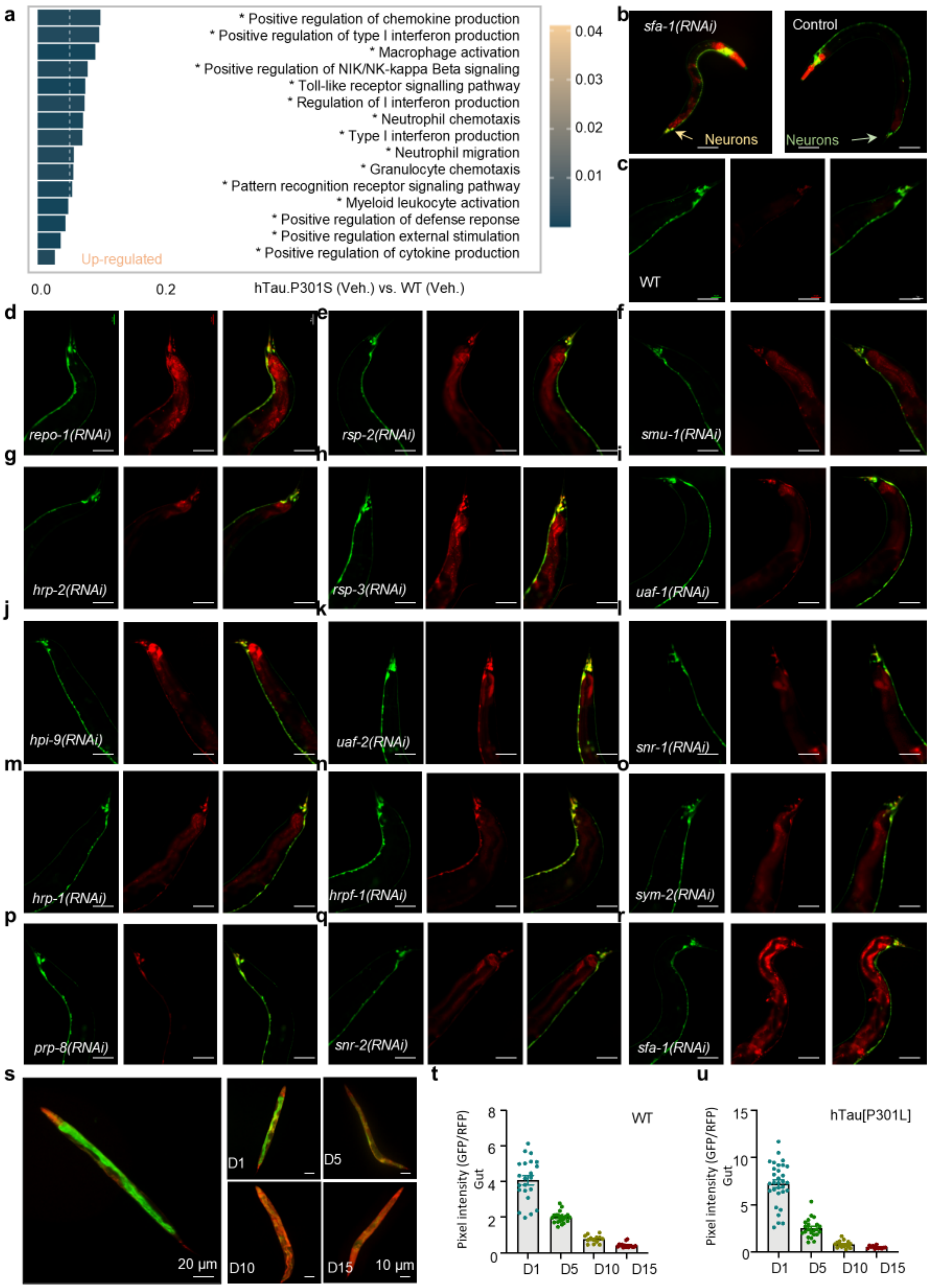

a
* Positive regulation of chemokine production
* Positive regulation of type I interferon production
* Macrophage activation
* Positive regulation of NIK/NK-kappa Beta signaling
* Toll-like receptor signalling pathway
* Regulation of I interferon production
* Neutrophil chemotaxis
* Type I interferon production
* Neutrophil migration
* Granulocyte chemotaxis
* Pattern recognition receptor signaling pathway
* Myeloid leukocyte activation
* Positive regulation of defense reponse
* Positive regulation external stimulation
* Positive regulation of cytokine production
Up-regulated
0.0
0.2
hTau.P301S (Veh.) vs. WT (Veh.)
0.04
0.03
0.02
0.01
b
sfa-1(RNAi)
Neurons
Control
Neurons
c
WT
d
repo-1(RNAi)
e
rsp-2(RNAi)
f
smu-1(RNAi)
g
hrp-2(RNAi)
h
rsp-3(RNAi)
i
uaf-1(RNAi)
j
hpi-9(RNAi)
k
uaf-2(RNAi)
l
snr-1(RNAi)
m
hrp-1(RNAi)
n
hrpf-1(RNAi)
o
sym-2(RNAi)
p
prp-8(RNAi)
q
snr-2(RNAi)
r
sfa-1(RNAi)
s
20 μm
D1
D5
D10
D15
10 μm
t
Pixel intensity (GFP/RFP) Gut
WT
D1
D5
D10
D15
u
Pixel intensity (GFP/RFP) Gut
hTau[P301L]
D1
D5
D10
D15

Fig. S1. Altered mRNA splicing in Tau pathology and aging.

a, Top 15 most statistically significant upregulated Gene Ontology (GO) term pathways enriched in the gene list of upregulated mRNA transcripts (Fisher's adjusted *p*-values < 0.05) in mice. **b**, Neuron-specific rgef-1 splicing in day 1 *C.elegans* feeding with (left) and without (right) *sfa-1* RNAi. **c-r**, Heterogeneous splicing patterns in response to knockdown of conserved splicing factors in *C. elegans*. Control (fed with L4440 RNAi) (**c**) and pan-neuronal knock-down of *repo-1* (**d**), *rsp-2* (**e**), *smu-1* (**f**), *hrp-2* (**g**), *rsp-3* (**h**), *uaf-1* (**i**), *hpi-9* (**j**), *uaf-2* (**k**), *snr-1* (**l**), *hrp-1* (**m**), *hrpf-1* (**n**), *sym-2* (**o**), *prp-8* (**p**), *snr-2* (**q**), and *sfa-1* (**r**) with RNAi from egg hatching, with images taken on day 3 of adulthood. **s**, A representative set of images of changes in RNA splicing in the gut tissue over time. **t, u,** Changes of splicing index (GFP/RFP) in gut between WT (**t**) and hTau[P301L] (**u**) worms of days 1, 5, 10, and 15.

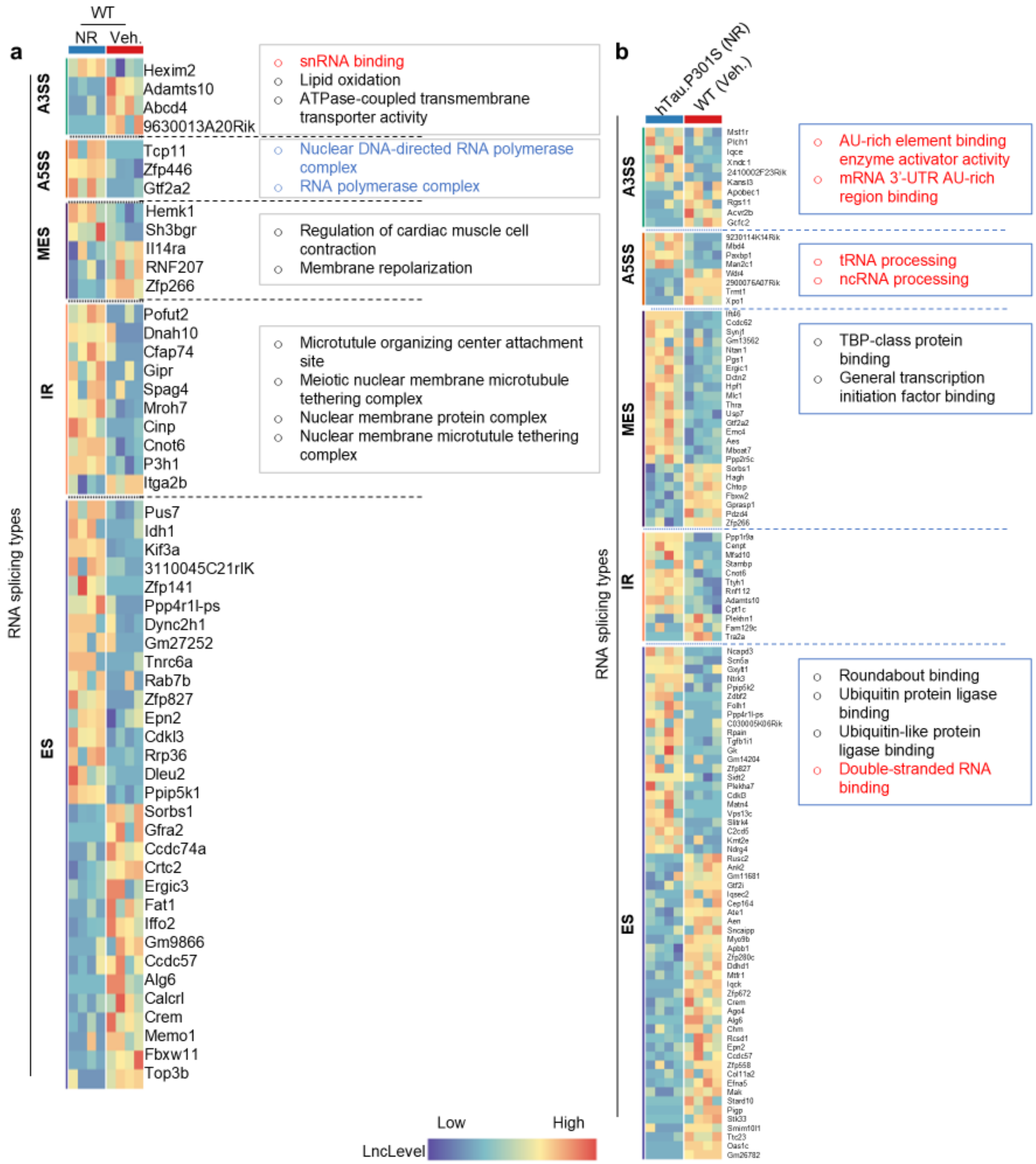

Fig. S2. Changes in alternative RNA splicing events in WT and hTau.P301S transgenic mice with/without the NAD$^+$ precursor NR.

a-b, Heatmaps comparing AS events based on alternative 3' splice site (A3SS), alternative 5' splice site (A5SS), multiple exon skip (MES), intron retention (IR), and exon skip (ES). Statistically significant Gene Ontology (GO) term pathways enriched in the gene list of changes in mRNA transcripts (Fisher's adjusted *p*-values < 0.05) in different types of alternative RNA splicing in mice. Heatmaps comparing AS events between WT mice treated with and without NR (**a**); data for hTau.P301S transgenic mice (NR) compared to WT (Veh.) shown in (**b**). Additional information shown in **Table S22, 23.**

a

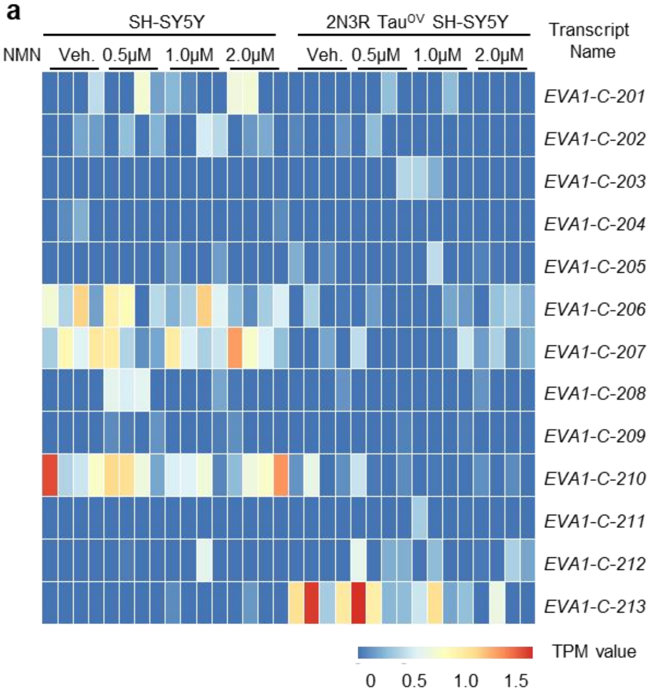

b

Name	ID	Length (bp)	Protein	Biotype
EVA1C -201	ENST0000 0300255.7	1671	441aa	Protein coding
EVA1C -202	ENST0000 0382699.7	1668	438aa	Protein coding
EVA1C -203	ENST0000 0401402.7	1558	393aa	Protein coding
EVA1C -204	ENST0000 0412833.1	936	153aa	Protein coding
EVA1C -205	ENST0000 0435323.5	1601	70aa	Nonsense mediated decay
EVA1C -206	ENST0000 0437338.5	1745	70aa	Nonsense mediated decay
EVA1C -207	ENST0000 0457807.5	1548	192aa	Nonsense mediated decay
EVA1C -208	ENST0000 0459833.5	687	No protein	Protein coding CDS not defined
EVA1C -209	ENST0000 0464037.5	2492	No protein	Retained intron
EVA1C -210	ENST0000 0469079.5	1689	No protein	Retained intron
EVA1C -211	ENST0000 0481638.1	384	No protein	Protein coding CDS not defined
EVA1C -212	ENST0000 0485488.1	720	No protein	Protein coding CDS not defined
EVA1C -213	ENST0000 0496615.5	492	No potein	Protein coding CDS not defined

c

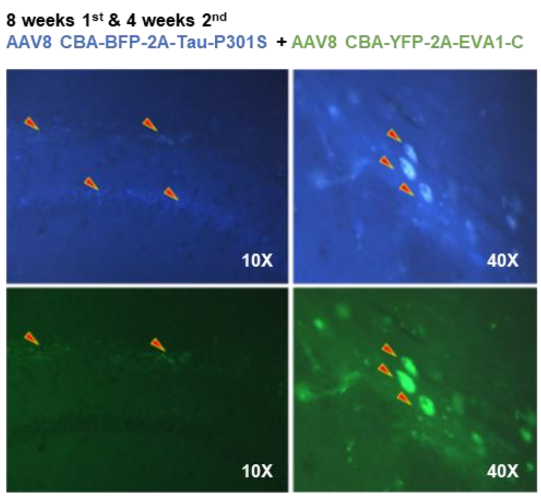

d

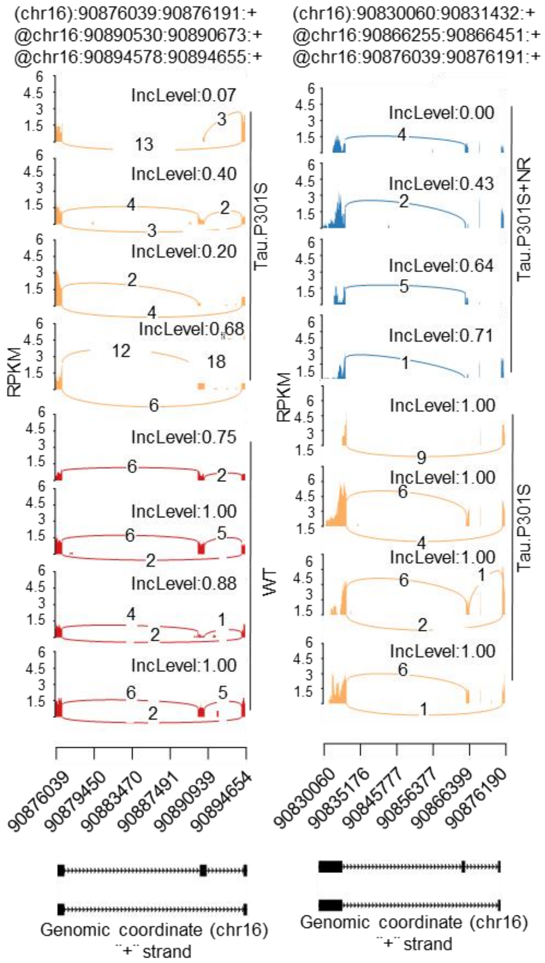

Fig. S3. NAD$^+$ changes the expression of EVA1C transcripts.

a, Heatmap showing different *Eva1C* transcripts expression in human SH-SY5Y and 2N3R tau-overexpressing SH-SY5Y with or without NMN at 4 different doses. **b**, Summary of biotype of *EVA1C* transcripts. **c**, The AAV efficiency in the WT mice 8 weeks after the injection of AAV8 CBA-BFP-2A-Tau P301S virus (blue), and 4 weeks after the injection of AAV CBA-YFP-2A-EVA1C virus (green); the arrowheads indicate cells the express both P301S Tau (blue) and EVA1C (green). **d**, Sashimi plot of EVA1C splicing changes in WT (Veh.), hTau.P301S (Veh.), and hTau.P301S (NR) groups. Diagrams on the left show the read coverage of exons. Plots on the right show the incLevel values that occurred in two paired tissues. The AS model of this region is represented in the lower panel. Each curve indicates the numbers of splicing sites and the number in the curve suggests the number of RNA-sequence reads in this region.

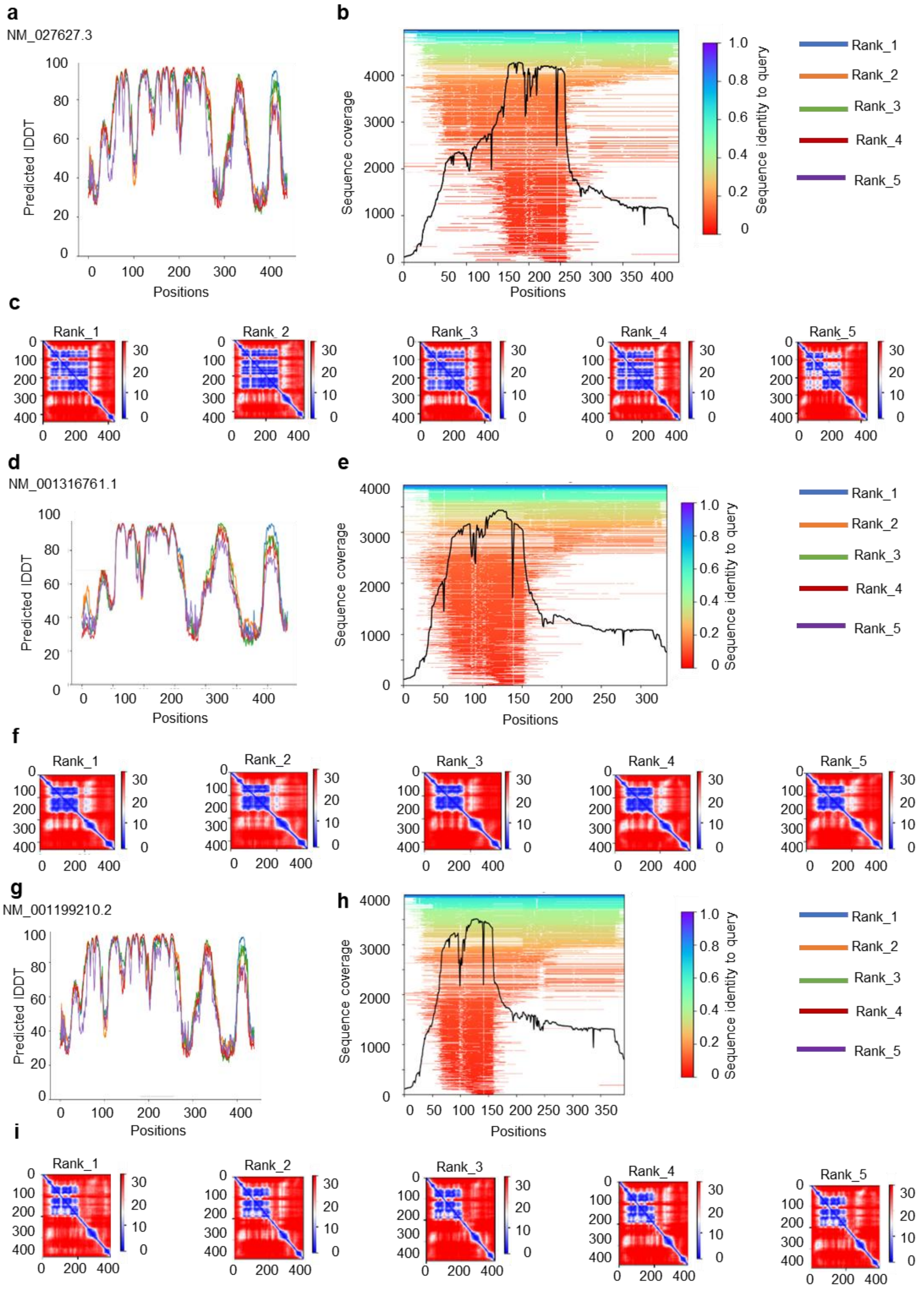
a
NM_027627.3
Predicted lDDT
Positions
b
Sequence coverage
Positions
Sequence identity to query
Rank_1
Rank_2
Rank_3
Rank_4
Rank_5
c
d
NM_001316761.1
e
f
g
NM_001199210.2
h
i

Fig. S4. Quality assessment of the tools used to predict EVA1C protein conformation.
a, d, **g,** *left* the IDDT (local Distance Difference Test score) plot which is a local superposition-free score associated with model confidence, while a higher IDDT score correlates with better performance of the model at that location. **b, e**, **h,** *right* the plot for the number of sequences per position. The criteria are: higher than 30 sequences per position, the three isoforms have over 30 sequences per position, and 93% of the sequence has over 100 reference sequences. **c, f, i,** the plot for predicted alignment error. This metric is applied to assess how confident the model is about the interface. The lower the score, the better.

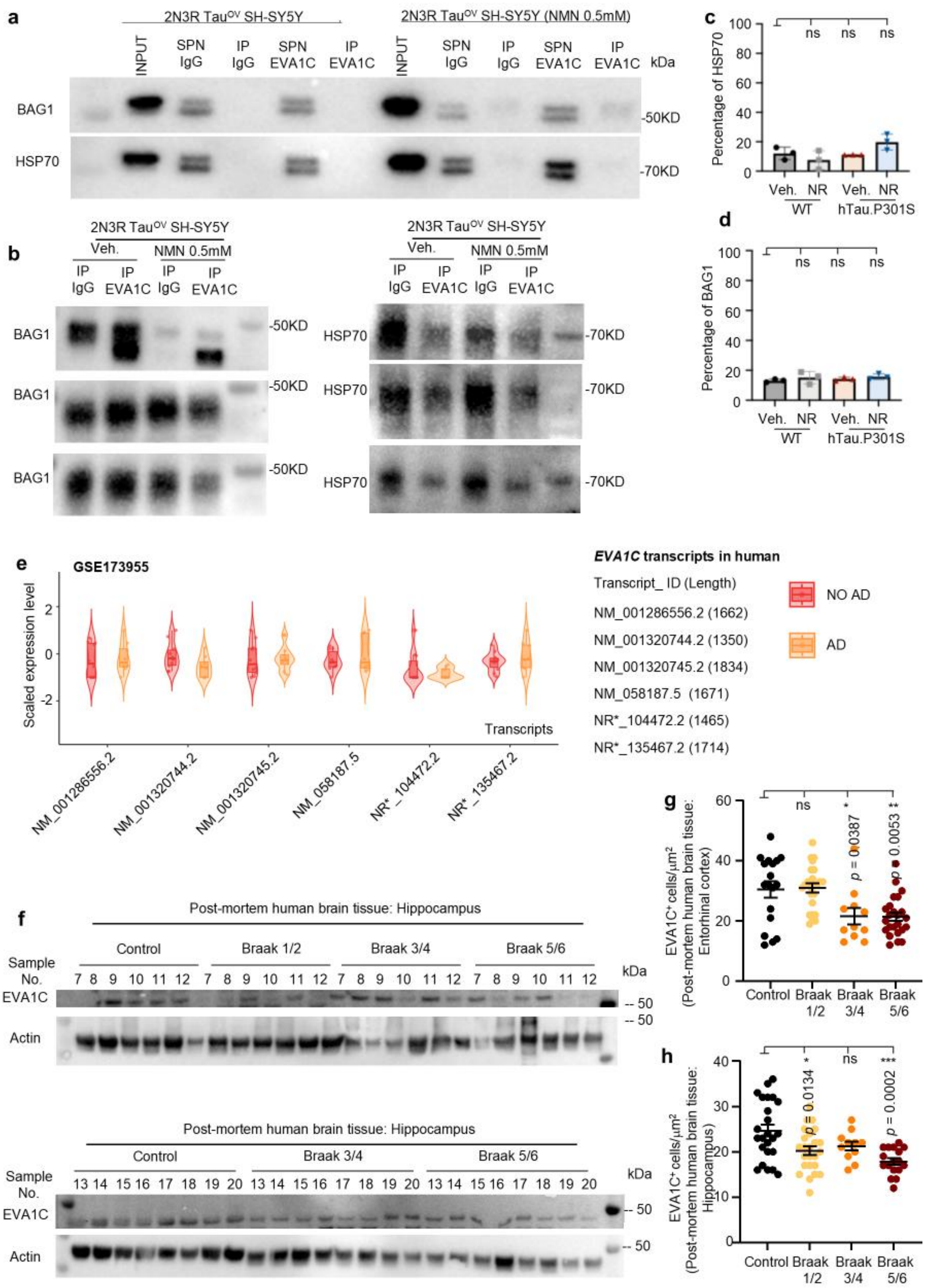

a
2N3R Tau^OV SH-SY5Y
2N3R Tau^OV SH-SY5Y (NMN 0.5mM)
INPUT
SPN IgG
IP IgG
SPN EVA1C
IP EVA1C
kDa
BAG1
HSP70
-50KD
-70KD
b
Veh.
NMN 0.5mM
-50KD
-70KD
c
Percentage of HSP70
ns
Veh. NR
WT
hTau.P301S
d
Percentage of BAG1
e
GSE173955
Scaled expression level
Transcripts
NM_001286556.2
NM_001320744.2
NM_001320745.2
NM_058187.5
NR*_104472.2
NR*_135467.2
EVA1C transcripts in human
Transcript_ ID (Length)
NM_001286556.2 (1662)
NM_001320744.2 (1350)
NM_001320745.2 (1834)
NM_058187.5 (1671)
NR*_104472.2 (1465)
NR*_135467.2 (1714)
NO AD
AD
f
Post-mortem human brain tissue: Hippocampus
Control
Braak 1/2
Braak 3/4
Braak 5/6
Sample No.
EVA1C
Actin
-- 50
g
EVA1C⁺ cells/μm² (Post-mortem human brain tissue: Entorhinal cortex)
p = 0.0387
p = 0.0053
h
EVA1C⁺ cells/μm² (Post-mortem human brain tissue: Hippocampus)
p = 0.0134
p = 0.0002

Fig. S5. The NAD$^+$-EVA1C axis regulates BAG-1 and HSP70 binding in human cells and Tau transgenic mice as well as reduced EVA1C protein level in postmortem tissues from AD patients and database compared to controls.

a, NMN treatment increases BAG1 and HSP70 protein expression in SH-SY5Y cells overexpressing 2N3R Tau. **b,** Co-immunoprecipitation (Co-IP) assays showing interactions of EVA1C with HSP70 and BAG-1. **c,d**, The percentages of HSP70 (C) and BAG-1 (D) in the designated groups. **e**, *EVA1C* transcripts identified in the human database GSE173955; **f**, Western blot data showing EVA1C protein expression in hippocampal brain tissues from AD patients with different Braak stages and age-matched healthy controls. Cognitively normal samples, *n*= 20; Braak 1/2, *n*= 12; Braak 3/4, *n*= 20; Braak 4/5, *n*= 20. **g,h**, The number of EVA1C$^+$ cells from AD patients at different Braak stages of disease and age-matched healthy controls (10 samples per group). Representative samples from entorhinal cortex (**g**) and hippocampal (**h**) brain regions were analyzed. Data are shown as mean ± s.e.m.; ns, no statistical significance; $^*p < 0.05$, $^{**}p < 0.01$, $^{***}p < 0.001$. Statistical significance was assessed by two-way or one-way ANOVA followed by Šidák’s multiple comparisons test as appropriate. All experiments were performed at least twice.

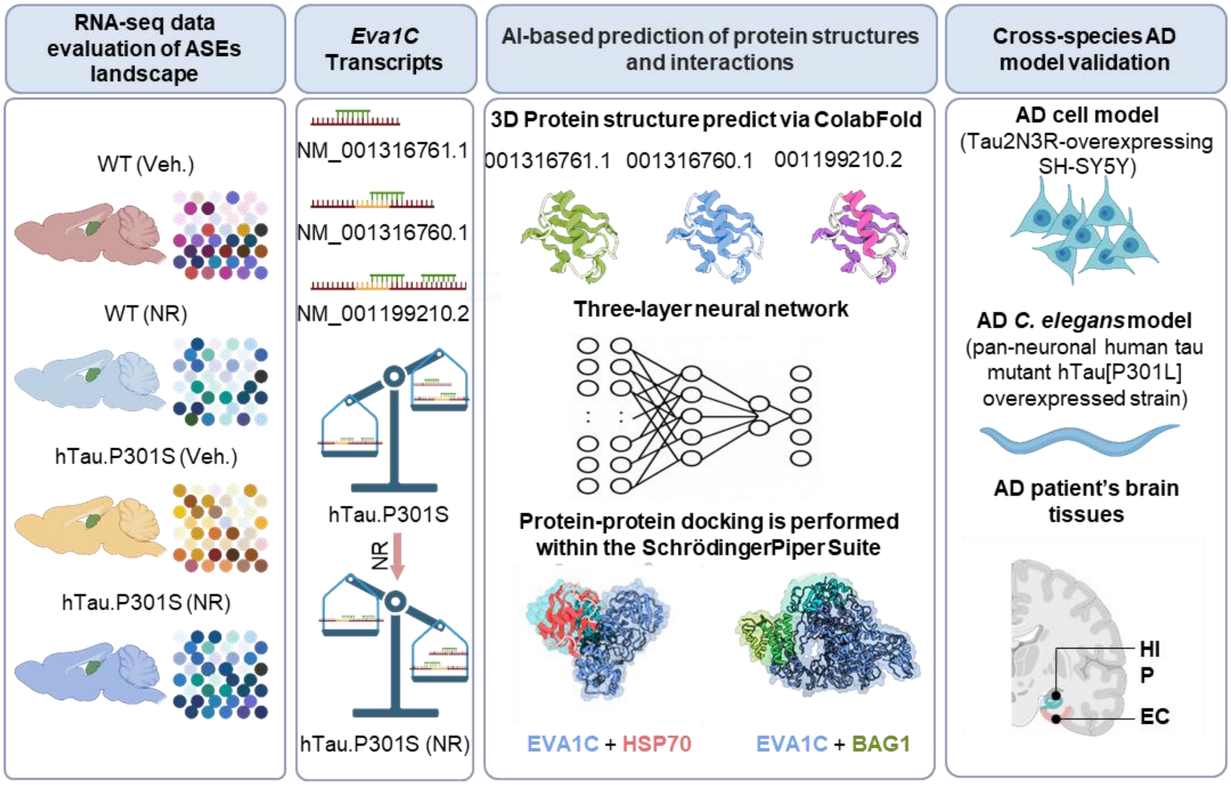

Fig. S6. An overview of the study design: a schematic summary illustrating the experimental workflow, including cross-species models, transcriptomic analyses, AI-based predictions, and data validation in *C. elegans*, cell, mouse, and human samples.

Table S4. A summary of splicing factors in *C. elegans* and their mammalian protein orthologs

***C. elegans* gene**	**Mammalian orthologues**	**Full name**	**Function**
uaf-1	*U2AF35*	U2 auxiliary factor small subunit	Core spliceosomal factor
uaf-2	*U2AF65*	U2 auxiliary factor large subunit	Core spliceosomal factor
sfa-1	*SF1/BBP*	Splicing factor 1, branch point binding protein	Core spliceosomal factor
repo-1	*SF3A2*	Splicing factor 3a subunit 2 (66kDa)	Core spliceosomal factor
snr-1	*SNRPD3*	Small nuclear ribonucleoprotein Sm D3	Core spliceosomal factor
snr-2	*SNRPB*	Small nuclear ribonucleoprotein Sm B	Core spliceosomal factor
rsp-2	*SRSF5, SRp40*	Serine/Arginine-rich splicing factor 5	Extrinsic non spliceosomal RNA binding protein
rsp-3	*SRSF1, SF2/ASF*	Serine/Arginine-rich splicing factor 1	Extrinsic non spliceosomal RNA binding protein
hrp-1	*hnRNP A1*	Heterogeneous nuclear ribonucleoprotein A1	Extrinsic non spliceosomal RNA binding protein
hrp-2	*hnRNP R*	Heterogeneous nuclear ribonucleoprotein R	Extrinsic non spliceosomal RNA binding protein
hrpf-1	*hnRNP F/H*	Heterogeneous nuclear ribonucleoprotein F/H	Extrinsic non spliceosomal RNA binding protein
prp-8	*PRPF8*	Pre-mRNA processing splicing factor 8	Core spliceosomal factor
phi-9	*NHP2L1*	NHP2-like protein 1	RNA binding protein
smu-1	*SMU1*	WD40 repeat-containing protein SMU1	RNA binding protein
sym-2	*hnRNP F/H*	Heterogeneous nuclear ribonucleoprotein F/H	Extrinsic non spliceosomal RNA binding protein

Table S18. A summary of the *C. elegans* lifespan data

Groups	Median lifespan (days)	Mean ± s.e.m. (days)	Statistics (p values)
WT (Veh.)	15.09	15.09 ± 0.5589	
hTau[P301L] (Veh.)	11.40	11.40 ± 0.3627	$p < 0.0001$ vs WT (Veh.)
hTau[P301L] (NR)	13.32	13.32 ± 0.4208	$p = 0.0015$ vs hTau[P301L] (Veh.) **
hTau[P301L]; *eva-1(RNAi)*$^{n\text{-}sid\text{-}1OV}$	11.78	11.78 ± 0.3634	$p = 0.6427$ vs hTau[P301L] (Veh.)
hTau[P301L]; *eva-1 (RNAi)*$^{n\text{-}sid\text{-}1OV}$ (NR)	11.36	11.36 ± 0.2618	$p = 0.4260$ vs hTau[P301L] (Veh.)

Table S19. A comparison of performance between our model and other PPI Prediction Models on STRING-HOMOSAPIENS

	STRING-HOMOSAPIENS	
	BFS	DFS
**Deep PPI**	56.68±1.0	66.82±0.3
**DNN-PPI**	53.05±0.8	64.94±0.9
**PIPR**	55.65±1.6	67.45±0.3
**ONTOPROTEIN**	70.59	81.94
**Our Model**	78.26±0.2	87.82±0.4

BFS: Breadth-First Search sampling; DFS: Depth-First Search sampling

Table S21. Detailed information of individuals with their postmortem brain tissues used in this study

Samples		Age	Sex	PMD (h)	Allele	Clinical Diagnosis	Tauopathy	Exp.
**No.**	**ID**							
**1**	A393/19	92	F	63	APOE 3/3	as control	Tau Braak stage 2	W
**2**	A078/17	98	F	76	APOE 3/3	as control	Tau Braak stage 2	W
**3**	A237/16	80	M	58	APOE 3/3	as control		W, I
**4**	A066/16	95	M	72.5	APOE 2/3	as control		W
**5**	A007/15	74	F	66	APOE 2/3	as control	Tau Braak stage 2	W, I
**6**	A319/14	90	F	44	APOE 3/3	as control	Tau Braak stage 2	W
**13**	A302/18	90	F	30	APOE 2/3	as control		W
**14**	A049/18	75	F		APOE 3/3	as control	Tau Braak stage 1	W
**15**	A226/17	90	F	48	APOE2/3	as control	Tau Braak stage 1	W
**16**	A382/16	87	M	48	APOE 3/4	as control		W
**17**	A297/16	81	M	10	APOE 3/3	as control	Tau Braak stage 1	W
**18**	A473/15	74	M	72	APOE3/4	as control		W
**19**	A242/15	82	M	26	APOE3/4	as control	Tau Braak stage 2	W
**20**	A234/15	80	M	34	APOE3/4	as control	Tau Braak stage 1	W
**1**	A142/16	83	M	48	APOE 2/3	Braak 1-2	Tau Braak stage 2	W, I
**2**	A046/14	74	F	72	APOE 3/3	Braak 1-2		W, I
**3**	A354/16	92	F	12	APOE3/3	Braak 1-2	Tau Braak stage 2	W
**4**	A073/05	93	M	33.00	APOE3/3	Braak 1-2	Tau Braak stage 2	W
**5**	A187/06	71	F	48	APOE3/4	Braak 1-2	Tau Braak stage 1	W
**6**	A067/09	92	F	19.5	APOE3/3	Braak 1-2	Tau Braak stage 3	W
**1**	A046/13	85	M	54	APOE 3/3	Braak 3-4	Tau Braak stage 3-4	W, I
**2**	A097/13	82	M	28	APOE4/4	Braak 3-4	Tau Braak stage 4	W, I
**3**	A097/15	90	F	43	APOE 3/3	Braak 3-4	Tau Braak stage 4	W
**4**	A266/15	90	F	82.5	APOE 3/3	Braak 3-4	Tau Braak stage 4	W
**5**	A065/16	91	M	48	APOE 2/4	Braak 3-4	Tau Braak stage 4	W
**6**	A444/18	95	M	61	APOE 2/3	Braak 3-4	Tau Braak stage 4	W
**13**	A374/14	88	M	79	APOE 3/4	Braak 3-4	Tau Braak stage 3-4	W
**14**	A233/13	92	M	70	APOE3/3	Braak 3-4	Tau Braak stage 4	W
**15**	A078/13	86	M	52.5	APOE3/4	Braak 3-4	Tau Braak stage 4	W
**16**	A357/11	91	M	28	APOE3/3	Braak 3-4	Tau Braak stage 4	W
**17**	A362/18	92	F	55	APOE 3/3	Braak 3-4	Tau Braak stage 4	W
**18**	A418/17	94	M	62	APOE 3/3	Braak 3-4	Tau Braak stage 4	W
**19**	A381/16	84	M	86	APOE 3/3	Braak 3-4	Tau Braak stage 4	W
**20**	A305/09	81	M	13	APOE 2/4	Braak 3-4	Tau Braak stage 3	W
**1**	A221/13	89	M	26	APOE3/4	Braak 5-6	Tau Braak stage 5	W
**2**	A355/14	79	F	31	APOE 3/3	Braak 5-6	Tau Braak stage 6	W, I
**3**	A366/14	82	F	68	APOE 3/4	Braak 5-6	Tau Braak stage 6	W
**4**	A377/14	85	F	79	APOE 4/4	Braak 5-6	Tau Braak stage 6	W

**5**	A395/14	92	M	36.5	APOE 4/4	Braak 5-6	Tau Braak stage 5	W, I
**6**	A156/15	86	F	43.5	APOE 4/4	Braak 5-6	Tau Braak stage 6	W
**13**	A258/16	67	M	39.5	APOE 4/5	Braak 5-6	Tau Braak stage 6	W
**14**	A166/16	89	M	21	APOE 3/2	Braak 5-6	Tau Braak stage 6	W
**15**	A087/16	89	F	38.5	APOE 3/3	Braak 5-6	Tau Braak stage 6	W, I
**17**	A163/15	76	F	4	APOE4/4	Braak 5-6	Tau Braak stage 6	W
**18**	A092/15	86	F	13	APOE3/4	Braak 5-6	Tau Braak stage 6	W, I
**19**	A342/14	84	F	27	APOE 3/4	Braak 5-6	Tau Braak stage 6	W
**20**	A118/20	87	F	44	APOE 3/3	Braak 5-6	Tau Braak stage 6	W
**1**	A141/18	82	F	42	APOE3/3	Control		I
**2**	A030/19	98	F	9	APOE3/3	Control	Tau Braak stage 1	I
**3**	A364/11	96	F	33	APOE3/4	Braak 1-2	Tau Braak stage 2	I
**4**	A344/16	77	M	96	APOE3/3	Braak 1-2	Tau Braak stage 2	I
**5**	A232/16	95	F	47	APOE3/4	Braak 3-4	Tau Braak stage 4	I
**6**	A084/16	86	F	55.5	APOE3/4	Braak 3-4	Tau Braak stage 4	I
**7**	A259/15	92	F	21	APOE4/4	Braak 5-6	Tau Braak stage 5	I

*** Western blotting (W), Immunostaining (I), postmortem delay (PMD), apolipoprotein E (APOE)**

Tables S1-S3, S5-S17, S20, S22, and S23 (Tables which are not listed here are in Excel)
Table S1: Differentially expressed genes in hTau.P301S versus WT mice

Table S2: GO enrichment analysis of differentially expressed genes in hTau.P301S mice

Table S3: RNA-seq analysis of alternative splicing events in hTau.P301S mice

Table S5: PCA results of transcriptional profiles in WT and hTau.P301S mice

Table S6: Differentially expressed genes and functional enrichment analysis in WT and hTau.P301S mice with or without NR

Table S7: Cluster analysis of gene expression profiles in WT and hTau.P301S mice with or without NR

Table S8: Classification of eight expression clusters into three classes-Cluster 8

Table S9: Pathway enrichment analysis of Class 1 clusters affected by NR in hTau.P301S mice

Table S10: Pathway enrichment analysis of Class 4 and 5 clusters affected by NR in hTau.P301S mice

Table S11: Pathway enrichment analysis of Class 8 clusters affected by NR in hTau.P301S mice

Table S12: Alternative splicing events (ASEs) identified in WT and hTau.P301S mice with or without NR treatment

Table S13: Distribution of alternative splicing event types across experimental groups

Table S14: ASEs specific to NR-treated hTau.P301S mice

Table S15: Transcripts with NR-modulated expression and splicing in hTau.P301S mice

Table S16: Effect of NAD^+ precursors (NR and NMN) on EVA1C mRNA abundance in worm and cell models

Table S17: Analysis of EVA1C protein biotypes in SH-SY5Y cells

Table S20: Predicted protein–protein interactions of three EVA1C isoforms

Table S22: Alternative RNA splicing events in different conditions (WT+NR vs. WT)

Table S23: Alternative RNA splicing events in different conditions (hTau.P301S+NR vs. WT)

Western blot full scans

a

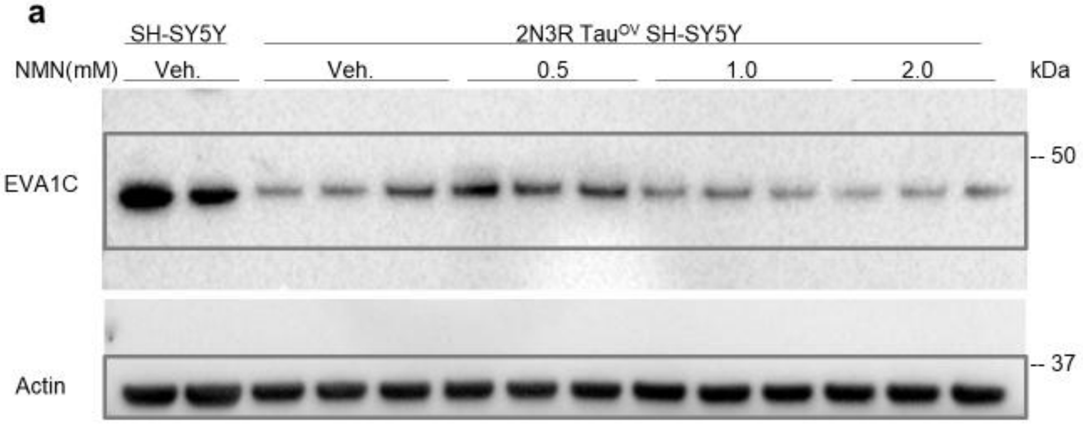

b

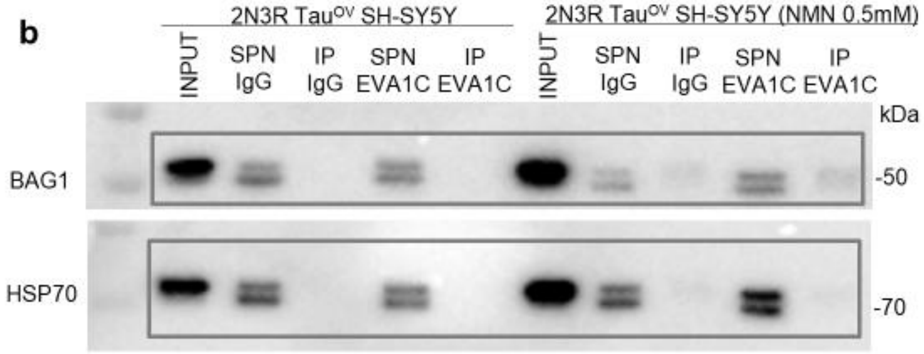

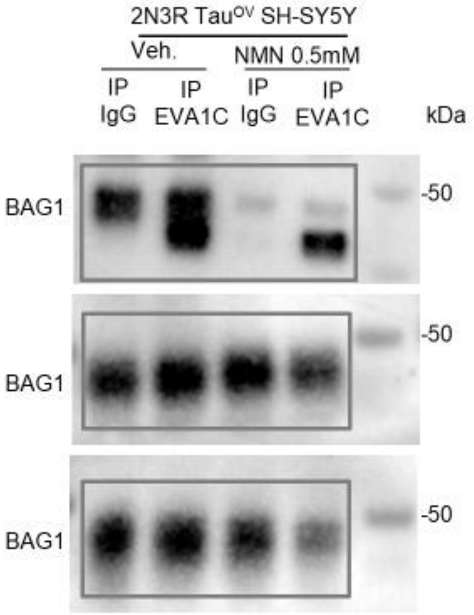

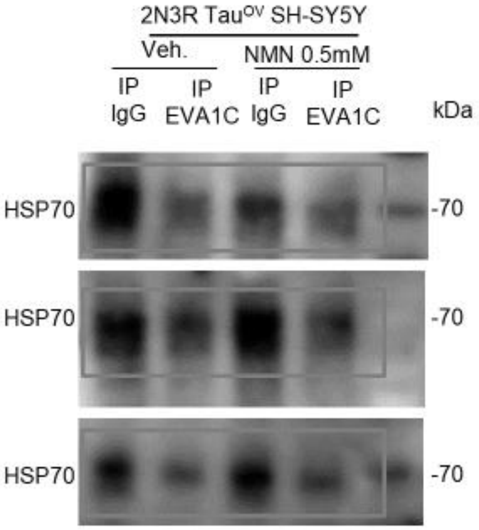

Source Data Fig.1

Western blot full scans

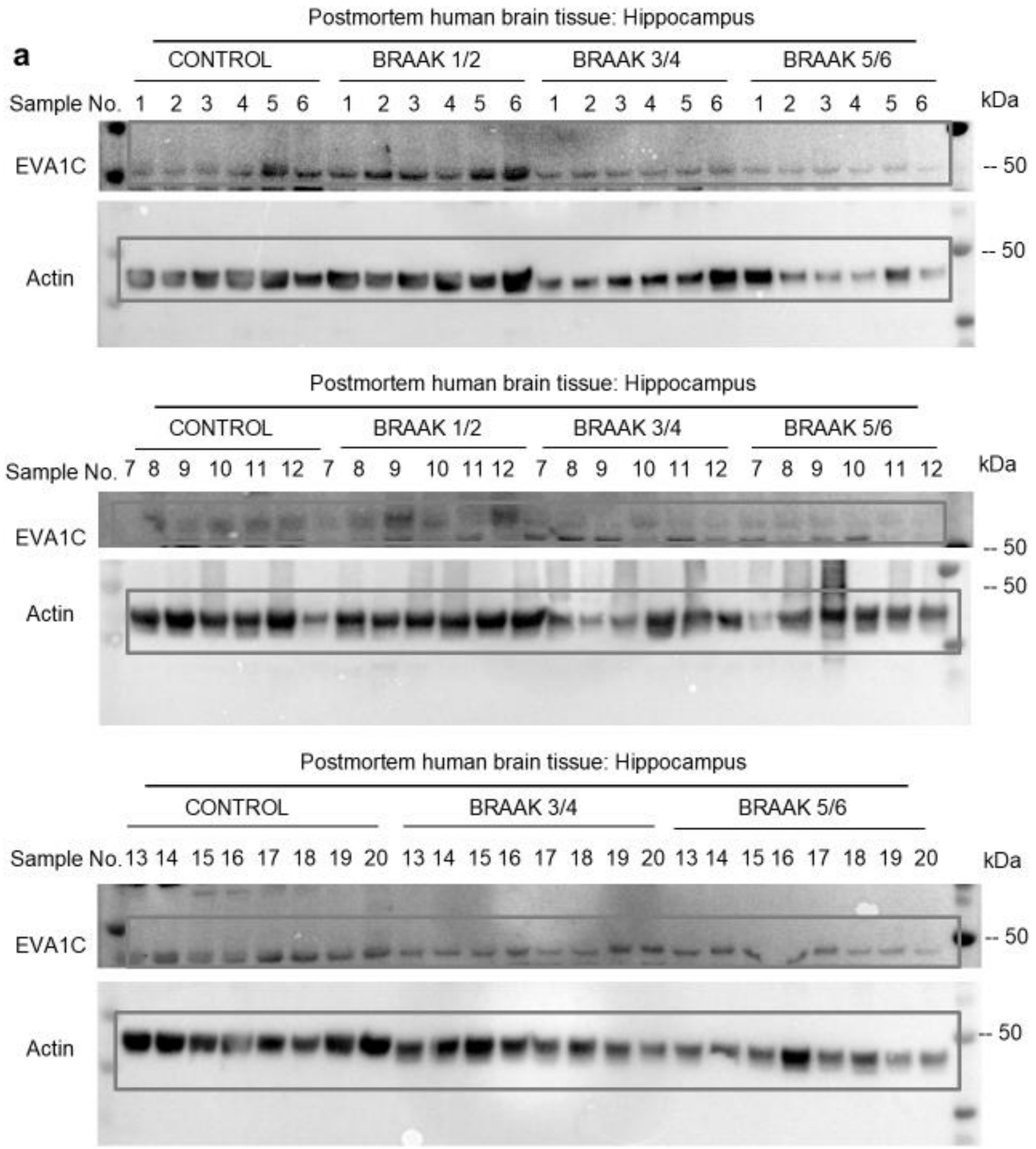

Source Data Fig.2
